# DNA Barcoding of Head Lice from a ~1500-Year-Old Salt-Preserved Mummy from the Chehrabad Salt Mine, Iran

**DOI:** 10.3390/insects17090929

**Published:** 2026-09-04

**Authors:** Negar Bizhani, Muhammad Ashfaq, Paul D. N. Hebert, Sean Prosser, Jean Dupouy-Camet, Abolfazl Ali, Thomas Stöllner, Nicole Boenke, Saied Reza Naddaf, Gholamreza Mowlavi

**Affiliations:** 1 Department of Medical Parasitology and Mycology, School of Public Health, Tehran University of Medical Sciences, Tehran 14155, Iran; nbizhani@ankara.edu.tr; 2 Center for Research of Endemic Parasites of Iran (CREPI), Tehran University of Medical Sciences, Tehran 14155, Iran; 3 Centre for Biodiversity Genomics, University of Guelph, Guelph, ON N1G 2W1, Canada; mashfaq@uoguelph.ca (M.A.); phebert@uoguelph.ca (P.D.N.H.); sprosser@uoguelph.ca (S.P.); 4 Faculté de Médecine, Paris Cité University, 15 Rue de l’École de Médecine, 75006 Paris, France; jean.dupouy-camet@orange.fr; 5 Archaeological Museum of Zanjan, Zanjan Cultural Heritage, Tourism, and Handicrafts General Directorate, Zanjan 4516773369, Iran; aaliabolfazl@gmail.com; 6 Institute for Archaeological Science, Ruhr-University Bochum, 44791 Bochum, Germany; thomas.stoellner@ruhr-uni-bochum.de (T.S.); nicole.boenke@rub.de (N.B.); 7 German Mining Museum, 44791 Bochum, Germany; 8 Department of Parasitology, Pasteur Institute of Iran, Tehran 1316943551, Iran

**Keywords:** lice, ancient DNA, mitochondrial DNA, phylogeny, genetic variation, DNA barcoding

## Abstract

Human lice can be classified into several mitochondrial clades with distinct geographic distributions, reflecting long-term coevolution with ancient hominids and possibly with human populations. Analysis of ancient lice DNA from archaeological human remains can provide insights into the geographic origins of these clades and the timing of their dispersal. This study analyzes the COI barcode region of lice recovered from the ~1500-year-old salt-preserved mummy (Salt Man 2) at the Chehrabad salt mine in Zanjan Province, Iran. The sequences were compared with those of recent lice from Iran and other regions, available in the GenBank database, to determine clade affiliation and possible historical relationships. Our phylogenetic analysis recovered the five recognized mitochondrial clades (A–E). Ancient lice clustered within clade A but displayed novel haplotypes. Recent nits from Gilan and Kurdistan provinces were grouped within clades A and B, the only clades reported in Iran. The most complete ancient sequence (MLICE005-18) showed close similarity to sequences from Iraq, Pakistan, Egypt, and Iran. These findings, along with future research, could help determine when ancient lice lineages were established or introduced into specific regions. They could also clarify whether they represent haplotypes that have since disappeared or a lineage that still exists in understudied human populations.

## Simple Summary

Human lice have lived alongside humans and their ancestors for thousands of years. Genetic data from lice can help scientists better understand human evolutionary history and migration. In this study, we examined ancient lice recovered from the hair of a naturally salt-preserved mummy who lived about 1500 years ago in what is now northwestern Iran. We compared their genetic data with those of recent lice from Iran and other parts of the world to assess their relationships. The ancient lice belonged to a widespread group still found in recent lice in Iran, but they also carried unique genetic variants not previously reported. Future studies using additional ancient and modern samples may reveal when different louse lineages became established in different regions and whether the ancient genetic variants have disappeared over time or persist in populations not yet studied. This research contributes to our understanding of the long-standing relationship between humans and their parasites.

## 1. Introduction

Lice (Insecta: Phthiraptera) are small, wingless ectoparasites that infest mammals and birds. Anoplura, one of the smallest suborders within the Phthiraptera order, comprises over 540 host-specific, blood-feeding species, suggesting long-term cospeciation with their hosts [1]. Primate lice are estimated to have coevolved with their hosts for at least 25 million years [2]. These tiny parasites have accompanied humans and other primates for millions of years and have played a notable role in human history [3]. Among blood-sucking lice, two species from two different genera infest humans: *Pediculus humanus* and *Phthirus pubis* (pubic “crab” lice) [4]. *P. humanus* exists in two morphotypes: *P. humanus* morphotype capitis (head lice) and *P. humanus* morphotype corporis (body lice). Head, body, and pubic lice inhabit different regions of the human body: head lice live on the scalp, body lice in clothing, and pubic lice in the pubic area [5]. Body lice evolved from head lice roughly 83,000 to 170,000 years ago, coinciding with the time when modern humans began wearing clothing made from animal hides. In contrast, pubic lice were acquired from gorillas about 3.3 million years ago, likely when early humans began losing their body hair as they moved out of the trees and onto the savannah [6].

Body lice serve as the primary vectors for several life-threatening human pathogens, including epidemic typhus, trench fever, and relapsing fever, caused by *Rickettsia prowazekii*, *Bartonella quintana*, and *Borrelia recurrentis*, respectively [7]. Additionally, combined epidemiological and laboratory studies have implicated head lice as potential vectors of human pathogens, although their vectorial capacity appears to be lower than that of body lice [8,9,10,11,12].

Human lice frequently feed on their specific host and cannot survive for an extended period without it, typically dying of starvation within 24 to 48 h [5]. However, recent evidence suggests that human lice can shift hosts and adapt to other closely related species to avoid extinction. This phenomenon of “host infidelity” may have allowed these obligate, host-specific parasites to persist despite the potential extinction of their primary hosts [2,4]. The host-shift phenomenon has provided a novel tool for researchers to study human evolution alongside the fossil record, archaeological findings, and recent genomic analyses.

DNA analyses of the human louse (*P. humanus*) from diverse geographic regions have revealed substantial sequence divergence, forming distinct mitochondrial clades that have been proposed to reflect long-term coevolution with different archaic hominins. The geographic distribution of these louse lineages supports the hypothesis that *Homo sapiens* coexisted with other hominin species in Asia and Europe. This has led to the proposal that modern humans may have acquired specific louse lineages through contact with archaic hominins, such as *Homo erectus*, Neanderthals, and Denisovans—after these species became extinct [13,14]. Recent analyses of nuclear genetic diversity further support using head lice as proxies for reconstructing human population history [15].

This study examines the standard cytochrome c oxidase subunit I (COI) gene of human lice recovered from a ~1500-year-old salt-preserved mummy, Salt Man 2, discovered in an ancient salt mine in Zanjan, Iran. It compares these lice with modern lice from Iran and with previously published reports in GenBank to determine their clade affiliation and explore their potential historical and geographical origins.

### 1.1. Study Area and Sample Collection

**Figure 1:**
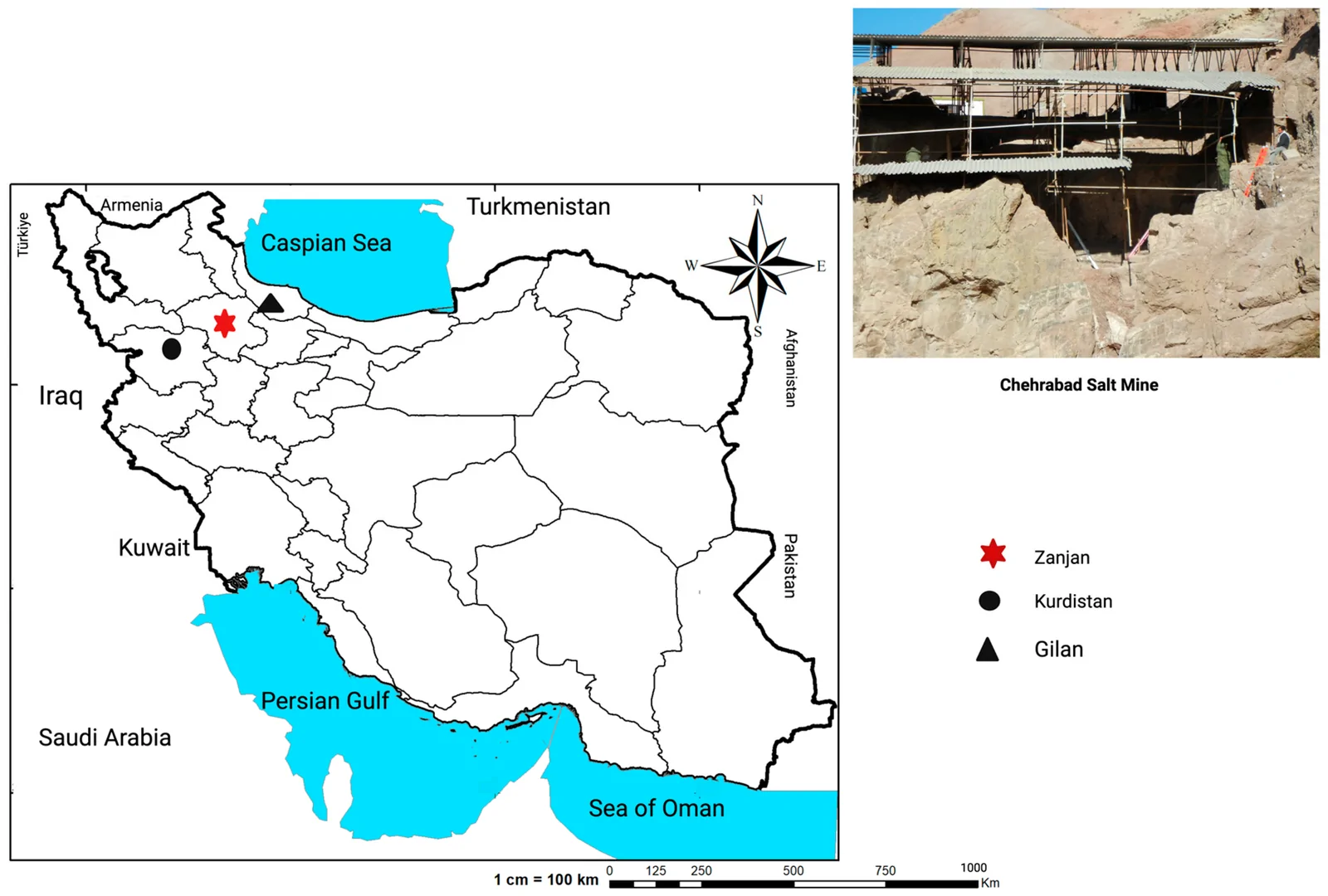
Left: Map of Iran showing the location of the Chehrabad salt mine in Zanjan Province, where ancient lice nits were recovered from Salt Man 2, along with Kurdistan and Gilan provinces, where modern lice nits were collected. Right: Archaeological excavation site at the Chehrabad salt mine. Created in BioRender. Bizhani, N. (2026). https://BioRender.com/9o1fk3q, accessed on 31 August 2026.

The Douzlākh (Azeri for “salty ground”) salt mine of Chehrābād (Chehrabad), hereafter the Chehrabad salt mine, is located in northwestern Iran, 75 km northwest of the city of Zanjan and approximately 1 km south of Hamzelou village, at an elevation of about 1350 m above sea level. Its geographic coordinates are 36°54′52″ N and 47°51′25″ E (Figure 1). Archaeological evidence indicates that the mine was exploited for salt during four periods: the Achaemenid era (6th–4th century BCE), the Sasanian era (3rd–7th century CE), and the middle and late Islamic periods, corresponding to the 11th–12th and 18th–20th centuries CE, respectively. Between 1993 and 2004, mining activities gradually ceased following the discovery of naturally salt-preserved human mummies, known as the Salt Men. Since 2009, the site has been protected under Iranian cultural heritage laws [16,17]. To date, the remains of eight Salt Men have been discovered, most of whom appear to have died when the mine galleries in which they were working collapsed [18,19]. These individuals represent rare examples from the ancient Persian period and are among the best-preserved salt mummies known worldwide.

**Figure 2:**
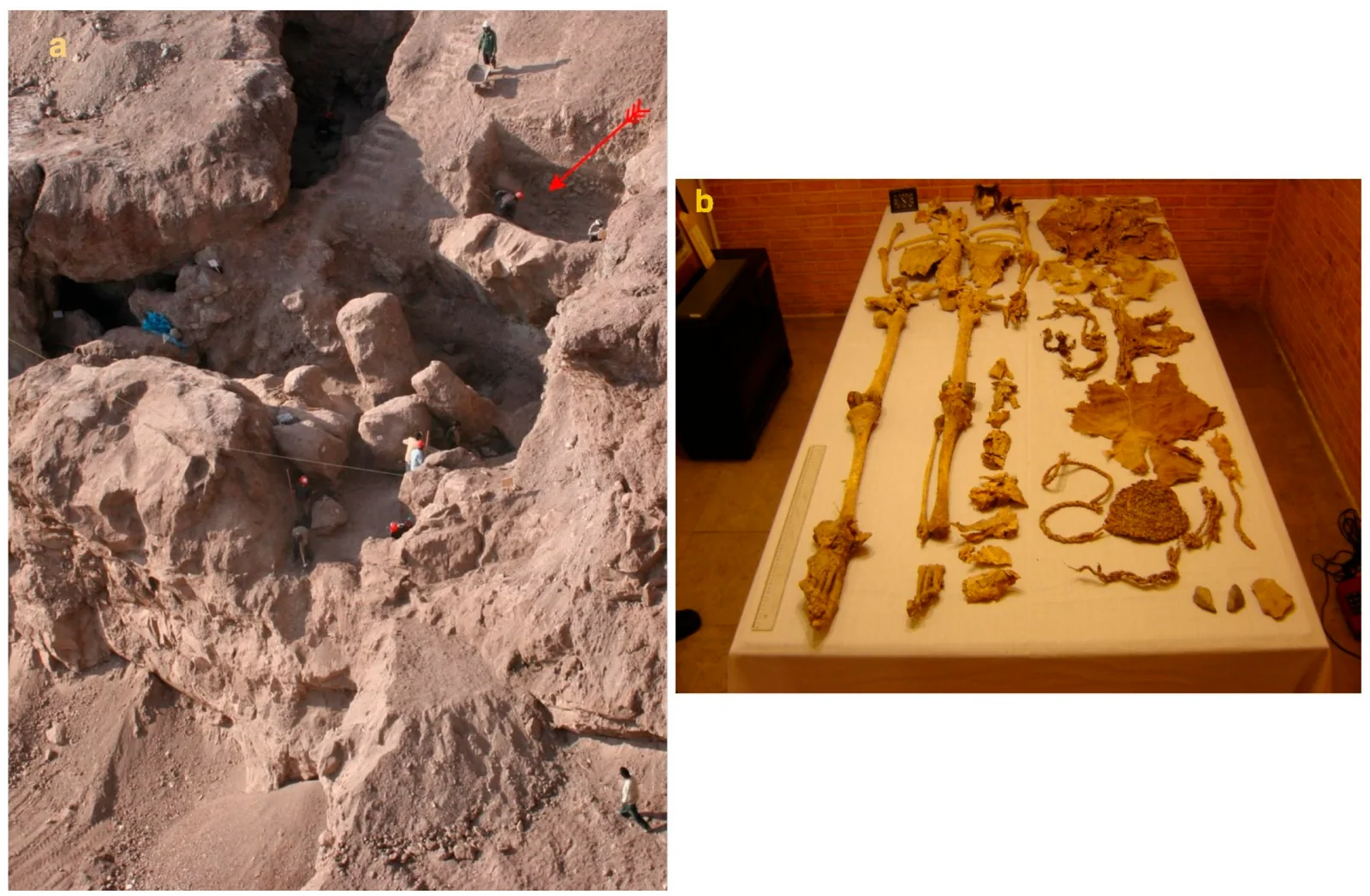
Overview of the Chehrabad salt mine in Zanjan Province, Iran, during archaeological excavations. (**a**) The red arrow marks the discovery site of Salt Man 2 [21] (image reproduced with permission from Abolfazl Ali). (**b**) Salt Man 2 and related artifacts.

Salt Man 2, one of the eight salt-preserved mummies, was accidentally discovered in 2004 when miners were bulldozing for salt and uncovered the remains in the upper excavation area [20] (Figure 2a). Unfortunately, the displacement of the remains by the bulldozers prevented the determination of its original in situ location, making stratigraphic dating impossible. Accelerator mass spectrometry radiocarbon (^14^C) dating indicates that Salt Man 2 and related artifacts date to the late Sassanian period, around 500–651 CE (1400–1500 years ago). However, a piece of fabric found beside the body was dated to the Late Arsacid–Early Sassanian transition (approximately 100–400 CE, 1600–1900 years ago) [21]. Salt Man 2, currently housed at the Zanjan Archaeology Museum in Iran, consists of a human skeleton with preserved skin, soft tissues, long head hair, and a beard on the jaw, indicating he was male (Figure 2b).

**Figure 3:**
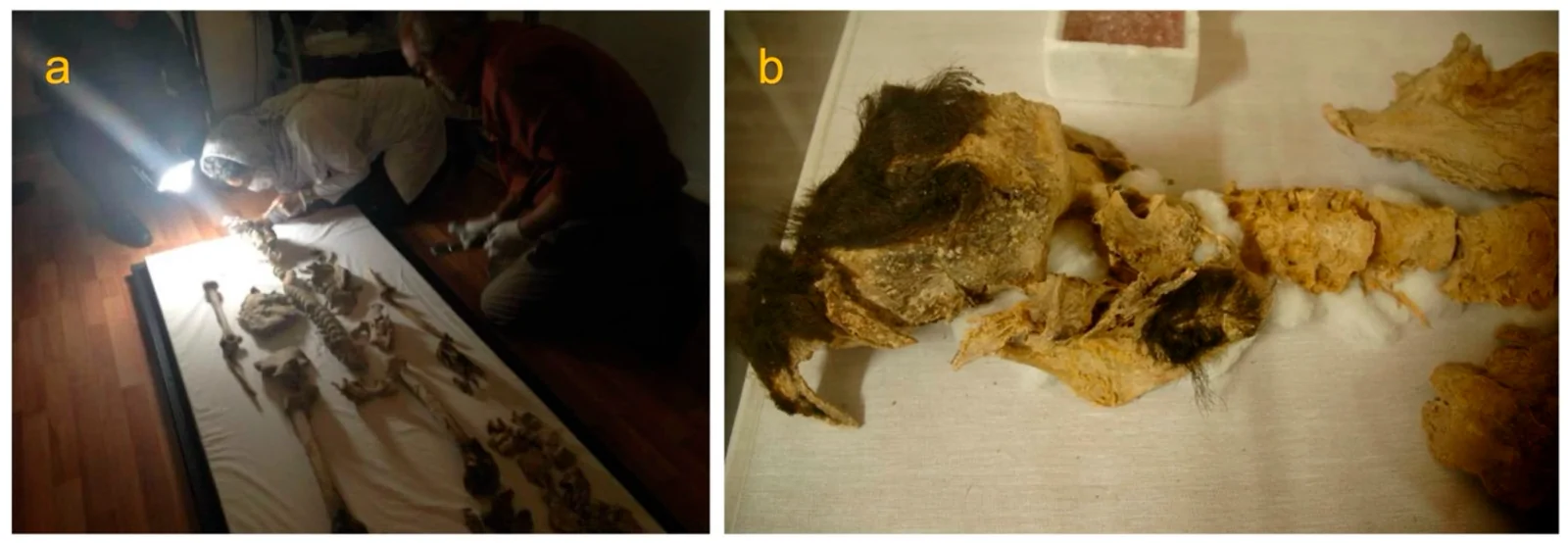
Collection of hair samples from Salt Man 2 at the Zanjan Archaeology Museum, Iran. (**a**) The complete remains of Salt Man 2’s body. (**b**) A close-up of the head with hair attached.

During a 2017 visit to the Zanjan Archaeology Museum, we observed a head lice infestation in Salt Man 2’s hair. With approval from the Ministry of Cultural Heritage, 40 hair strands were carefully cut using fine scissors, 29 of which contained nits (Figure 3).

**Figure 4:**
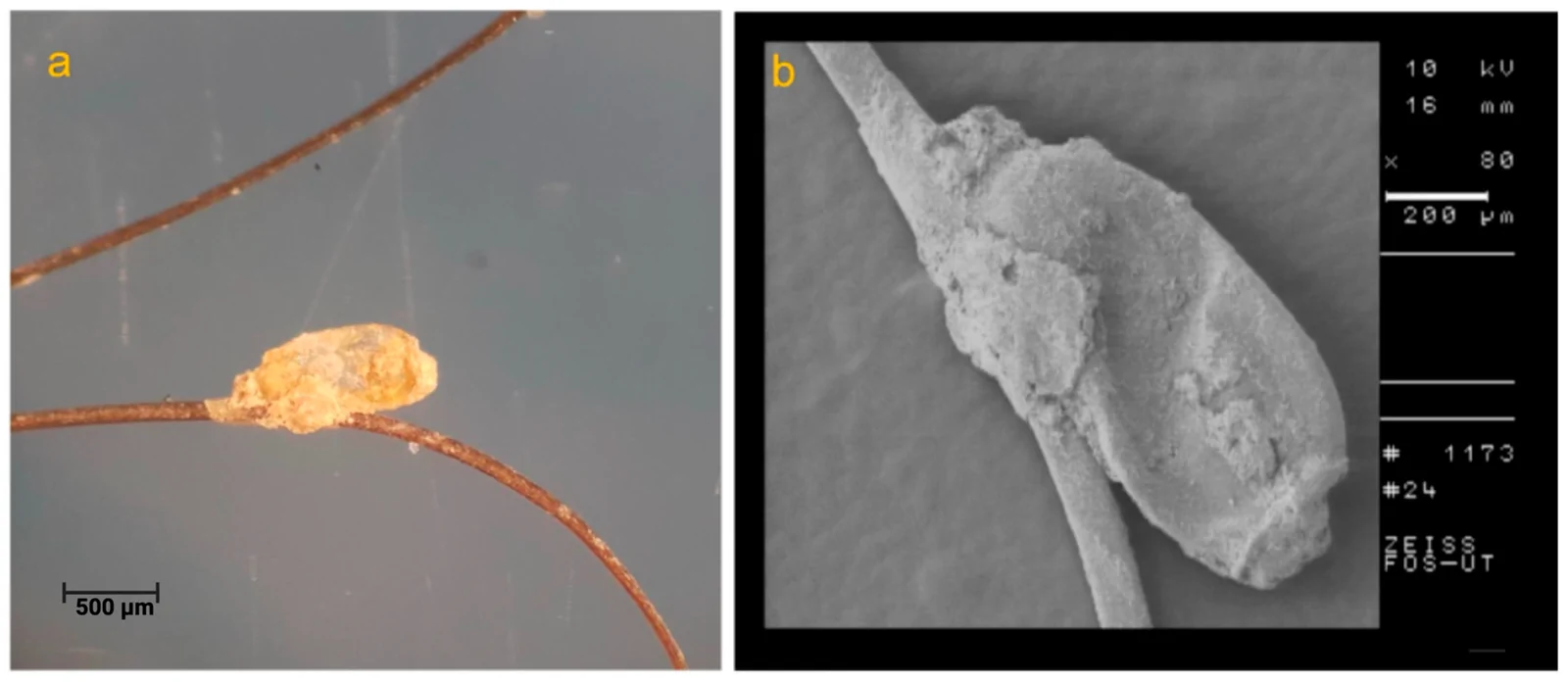
An ancient nit attached to a single strand of hair from Salt Man 2, a salt-preserved mummy kept at the Zanjan Archaeology Museum, Iran. (**a**) Viewed under a stereomicroscope. (**b**) Viewed under a scanning electron microscope (SEM) (Zeiss LEO EM906, Oberkochen, Germany).

The nits were subsequently photographed under light microscopy and scanning electron microscopy (SEM) (Figure 4).

Recent nits were collected from female students in Gilan and Kurdistan provinces (northern and western Iran) during routine head-lice inspections in primary schools (Figure 1). These samples were collected in 2017 as part of an MSc thesis, with approval from the Ethics Committee of Tehran University of Medical Sciences and with informed verbal consent from all participants or their guardians, who were fully informed about the study before participating. Complete anonymity was maintained for each contributor as part of the informed consent process.

Both ancient and contemporary nit samples were sent to the Canadian Centre for DNA Barcoding (CCDB), Centre for Biodiversity Genomics, University of Guelph, Canada (https://ccdb.ca, accessed on 20 June 2018), for molecular analysis. Nits on human hair were received at CCDB in two shipments, each sealed in a courier envelope: the first included nine ancient lice nits from Salt Man 2, and the second contained 13 recent lice nits. All samples were stored at −20 °C until DNA extraction.

### 1.2. DNA Extractions

DNA extraction and sequencing were performed at CCDB. All ancient nit samples were processed in a dedicated clean laboratory for degraded samples. Equipment and surfaces were sterilized with ultraviolet light before and after processing, and all reagents were freshly prepared. Personnel wore gloves, full-body suits, hoods, and face masks to prevent contamination. Three negative controls without nits were included in each extraction. Tubes were opened under sterile conditions, and nits were photographed before being transferred into clean 1.5 mL tubes containing 200 µL of lysis buffer (700 mM guanidine thiocyanate (Sigma-Aldrich, St. Louis, MO, USA), 30 mM Tris-HCl, pH 8.0 ((Sigma-Aldrich, St. Louis, MO, USA), 30 mM EDTA, pH 8.0 (Thermo Fisher Scientific, Waltham, MA, USA), 0.5% Triton X-100 ((Sigma-Aldrich, St. Louis, MO, USA), 5% Tween-20 (Sigma-Aldrich, St. Louis, MO, USA), and 2 mg/mL proteinase K (Promega, Madison, WI, USA). Samples were incubated at 56 °C for 18 h with gentle shaking. Next, 400 µL of binding mix (3 M guanidine thiocyanate, 5 mM Tris-HCl pH 6.4, 10 mM EDTA pH 8.0, 2% Triton X-100, 50% ethanol) was added, and suspensions were applied to silica membrane spin columns (Epoch Biolabs, Inc., Missouri City, TX, USA) and centrifuged at 6000× *g* for 2 min. Membranes were washed sequentially with 180 µL protein wash buffer (1.56 M guanidine thiocyanate, 2.6 mM Tris-HCl pH 6.4, 5.2 mM EDTA pH 8.0, 1.04% Triton X-100, 70% ethanol) and 700 µL wash buffer (50 mM NaCl, 10 mM Tris-HCl pH 7.4, 0.5 mM EDTA pH 8.0, 60% ethanol), and centrifuged at 6000× *g* for 2 min and 10,000× *g* for 4 min, respectively. Membranes were dried at 10,000× *g* for 4 min and incubated at 56 °C for 30 min. DNA was eluted with 40 µL sterile elution buffer (10 mM Tris-HCl, pH 8.0), incubated for 1 min at room temperature, centrifuged at 10,000× *g* for 5 min, transferred to clean tubes, and stored at −20 °C.

Recent nits were processed in a separate, physically isolated lab at the CCDB using the same protocol under standard sterile conditions, only after all ancient samples had been processed.

### 1.3. COI Amplification from Ancient Nits

Sixteen primers were designed to amplify seven short, specific, overlapping fragments spanning the *P. humanus* standard COI barcode region (Table 1).

In *P. humanus*, the mitochondrial genome is uniquely organized into 20 small circular minichromosomes [23]. The COI gene spans a 1572-bp coding sequence and is located on the mitochondrial minichromosome that carries this gene (GenBank accession: HM357247.1). This study targets the standard 658-bp barcode region at the 5′ end of the gene, corresponding to positions 71–729 of the coding sequence [24]. This region corresponds to the conserved Folmer fragment, widely used for species identification [25]. All primers show 100% identity across the five reported *P. humanus* clades, and all amplicons were designed to be shorter than 120 bp (101–118 bp) to accommodate ancient DNA degradation.

The 12 µL reactions contained 1.62 µL of molecular-grade water (Cytiva, HyClone Laboratories, Inc., South Logan, UT 84321, USA), 6.25 µL of 10% trehalose (Thermo Fisher Scientific, Waltham, MA, USA), 1.25 µL of Platinum Taq 10x buffer (Thermo Fisher Scientific, Waltham, MA, USA), 0.625 µL of 50 mM MgCl_2_ (Thermo Fisher Scientific, Waltham, MA, USA), 0.0625 µL of 10 mM dNTPs (KAPA Biosystems, Wilmington, MA, USA), 0.125 µL each of 10 µM forward and reverse primers (IDT), 0.06 µL of 5 U/µL Platinum Taq (Thermo Fisher Scientific, Waltham, MA, USA), and 2 µL of template DNA. Amplifications were consistent across all seven reactions and included enzyme activation at 95 °C for 2 min, followed by 60 cycles of 95 °C for 40 s, 45 °C for 40 s, and 72 °C for 30 s, with a final extension at 72 °C for 2 min. PCR success was confirmed by analyzing 4 µL of the amplicons on a 2% agarose E-Gel 96 system (Invitrogen, Carlsbad, CA, USA).

### 1.4. COI Amplification from Recent Nits

The 658-bp COI barcode region [26] was amplified from recent lice using the forward and reverse cocktails, which consisted of equimolar mixtures of the insect primers LepF1/LepR1 and general Folmer primers LCO1490/HCO2198 [22] in 12.5 μL reactions [27] containing 2 μL of DNA template. Thermocycling involved enzyme activation at 95 °C for 2 min, followed by 5 cycles of 95 °C for 40 s, 45 °C for 40 s, and 72 °C for 1 min. This was then followed by 35 cycles of 95 °C for 40 s, 51 °C for 40 s, and 72 °C for 1 min, ending with a final extension at 72 °C for 5 min. The amplifications were visualized as described above.

### 1.5. COI Sequencing and BLAST Analysis

PCR products (~8 µL) with a visible band on an E-Gel were diluted with 20 µL of sterile water, while those without a visible band were used undiluted. These PCR products served as templates for Sanger sequencing on an ABI 3730xl DNA Analyzer using a modified BigDye Terminator v3.1 Cycle Sequencing Kit (Thermo Fisher Scientific, Waltham, MA, USA). Cycle sequencing reactions of 9.5 µL were prepared with standard CCDB reaction mixes containing 0.25 µL of BigDye Terminator v3.1 dye mix (Thermo Fisher Scientific, Waltham, MA, USA), 1.875 µL of 5× sequencing buffer (Thermo Fisher Scientific, Waltham, MA, USA), 5 µL of 10% trehalose (Thermo Fisher Scientific, Waltham, MA, USA), 1 µL of either forward or reverse 10 µM sequencing primer (Integrated DNA Technologies, Coralville, IA, USA), 1 µL of PCR product, and 0.975 µL of molecular-grade water (Cytiva, HyClone Laboratories, Inc., South Logan, UT 84321, USA).

Sequence trace files (Supplementary data S1) were manually edited, and overlapping reads were assembled into contigs. The assembled sequences were compared with sequences available in GenBank using the NCBI Basic Local Alignment Search Tool (BLAST) to assess sequence identity and identify the closest matches. The assembled contigs were then uploaded to the Barcode of Life Data System under the project MLICE (https://boldsystems.org/, accessed on 13 August 2018).

### 1.6. GenBank Submission

All sequences were submitted to the GenBank database under accession numbers PV426995–PV427007.

### 1.7. Sequence Alignment and Tree Construction

Sequences from both recent and ancient samples generated in this study, along with sequences representing clades A, B, C, D, and E in the GenBank database, were aligned using the CLUSTAL W algorithm implemented in MEGA 7.0.26 [28,29]. We selected representative sequences to (1) include all major COI clades reported previously and (2) incorporate sequences with high similarity to our samples, which enhances phylogenetic resolution within clades A and B. The phylogenetic analysis was conducted with MEGA7.0.26 [29], and phylogenetic trees were constructed using the neighbor-joining method with the pairwise deletion option and the maximum likelihood algorithm based on the best-fit model, specifically the Tamura-3-parameter (T92) model [29]. Bootstrap values were calculated with 1000 replicates. The resulting trees were rooted with the midpoint method and visualized using FigTree v.1.4.4.

## 2. Results

### 2.1. COI Amplification and Sequencing

Of the nine ancient samples, only three yielded enough overlap amplifications to sequence the COI barcode region (Supplementary data S2). The contigs from samples MLICE005-18 and MLICE007-18 measured 658 bp and 478 bp, respectively. In contrast, the contig from sample MLICE004-18 had over 50% of nucleotide reads missing and was excluded from further analysis. (Supplementary data S3, Table 2). Of the 13 recent nits, 11 produced the expected PCR band, including six from Gilan and five from Kurdistan (Table 2). Sequencing of recent nit samples yielded fragments ranging from 557 to 658 nucleotides.

### 2.2. BLAST Analysis

The recent nits from Gilan were identical and showed 100% sequence identity to isolates from Iraq (accession numbers MK913649, OP164668, OP164667, OP164666, and OP164662) across the entire sequence. The recent nits from Kurdistan comprised two haplotypes differing by a single nucleotide substitution (T → C at position 43). One haplotype, represented by isolates L-W-2 and L-W-5, showed 100% sequence identity to isolates from South Africa and Finland (accession numbers KJ839921 and MZ608883) over 99–100% query coverage. The second haplotype, represented by isolates L-W-3, L-W-6, and L-W-7, was novel and shared 99.85% sequence identity with the same South African and Finnish isolates.

The two ancient isolates, MLICE005-18 and MLICE007-18, shared 98.33% sequence identity across the 478-bp overlapping region, which represents 73% of the full-length COI barcode (478/658 bp). MLICE005-18 showed 99.70% sequence identity to isolates from Iraq, Australia, and Egypt (accession numbers ON183986, HM352244, HM352246, HM352247, and KJ839870). In contrast, MLICE007-18 showed the highest sequence identity (98.95%) to an isolate from Pakistan (accession number KJ839858).

### 2.3. Phylogeny

The phylogenetic trees inferred using the NJ and ML methods showed nearly identical topologies. Therefore, we present only the ML tree. Notably, the definitions of clades D and E differ across previous studies [14,25]. Here, we follow the more recent classification proposed by Amanzougaghene [14].

**Figure 5:**
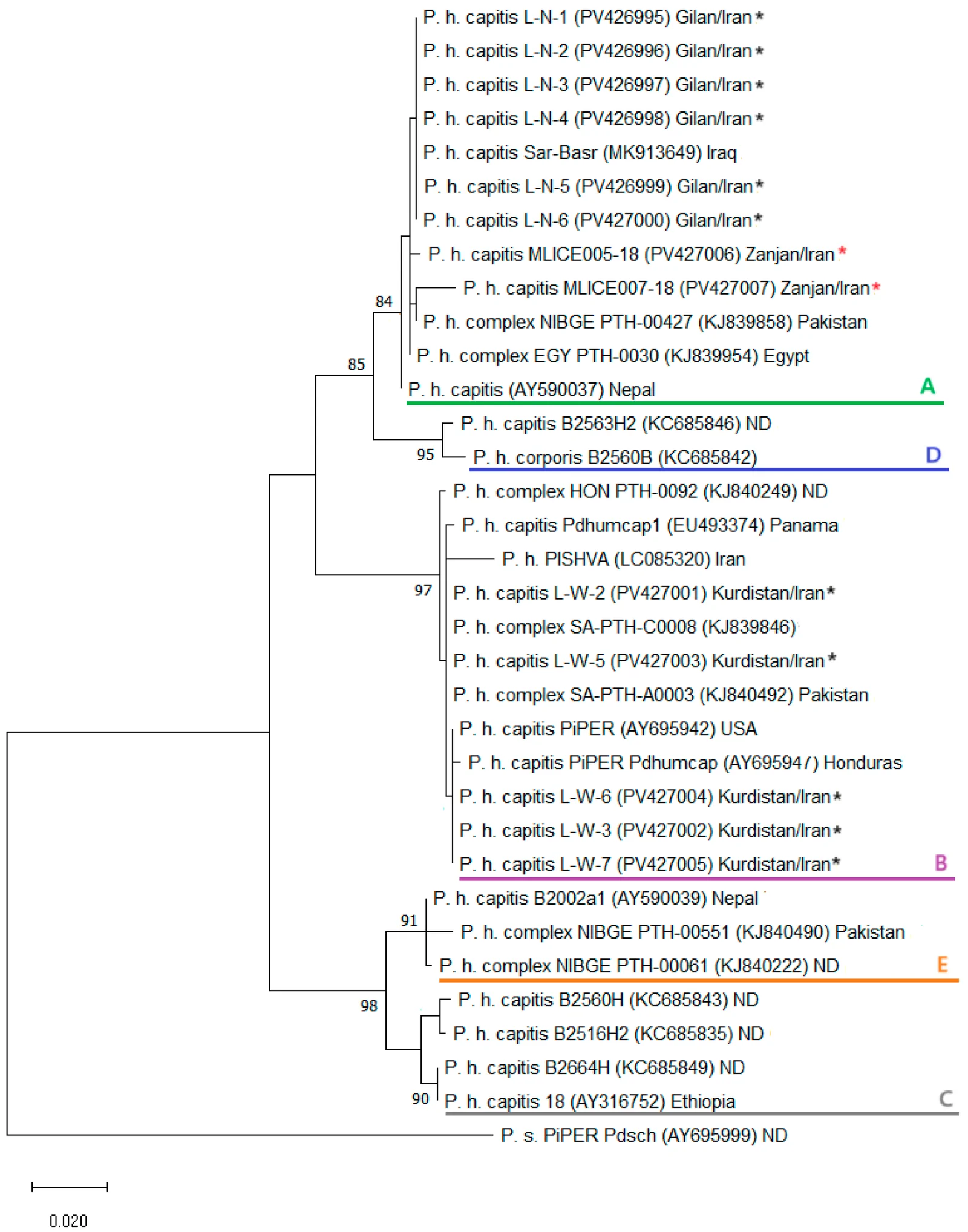
ML phylogenetic tree based on the standard 658-bp COI barcode region. A–E represent different lice clades. Black and red asterisks indicate sequences from contemporary and ancient lice, respectively. The numbers at the nodes indicate the bootstrap probabilities; support values below 70 were omitted for clarity. The scale bar indicates 0.02 estimated nucleotide substitutions per site.

The sequences clustered into five mitochondrial clades (A–E). The recent nit sequences from Gilan and Kurdistan were assigned to clades A and B, respectively. The six recent nit sequences from Gilan clustered with the Iraq (Sar-Basr) sequence within clade A, whereas the recent nit sequences from Kurdistan clustered with isolates from Panama, Pakistan, the USA, Honduras, and a previously reported sequence from Iran (Figure 5).

The ancient nit sequences were assigned to clade A. One ancient nit (MLICE005-18) formed a distinct lineage within this clade, whereas the other (MLICE007-18) was sister to the Pakistan isolate (accession no. KJ839858) (Figure 5).

In a broader phylogenetic tree, which included all available GenBank sequences, several clades were further resolved into multiple subclades (Supplementary data S4).

## 3. Discussion

Mitochondrial DNA (mtDNA) is maternally inherited. It rarely undergoes recombination, making it a valuable marker for tracing ancestral polymorphisms and preserving genetic signatures that reflect the long-term evolutionary history of human lice. Complementing mitochondrial analyses, recent studies of nuclear genetic diversity have shown that head lice also retain signatures of major human dispersal events, further supporting their value as proxies for reconstructing human population history [15].

Phylogenetic analyses of mitochondrial genes have identified five divergent mitochondrial clades (A–E) using the COI gene and six clades (A–F) using the mitochondrial cytochrome *b* (*cytb*) gene in human lice worldwide [14,25,30,31,32,33,34]. Head lice have been reported in all recognized mitochondrial clades, whereas body lice are restricted to clades A and D [30,31,35].

The divergence of *P. humanus* into different clades dates back about 2 million years, predating the emergence of modern humans by about 0.2 million years. This variation among clades suggests a complex evolutionary history involving coevolution with different hosts, including now-extinct archaic hominins in distinct geographical regions [35,36].

The exact origins and migration routes of human lice clades are still debated and may change as discoveries are made. While genetic information from ancient human lice offers valuable insights into past distributions, some theories remain contested. For example, the discovery of clade B, first described in modern lice from the Americas [2,33,35], found in pre-Columbian mummies, led to a hypothesis of an American origin [37]. Later, detecting this clade in 2000-year-old lice from the Middle East challenged this idea and supported an Asian origin [31]. More recently, the discovery of clade F in Amazonia, which diverged from clade B as a recent sister lineage, supports a biogeographic history consistent with an American origin [14]. This ongoing debate highlights the complexity of lice migration and their links to human movement.

In this study, we successfully amplified and sequenced the standard COI barcode region from two nits recovered from the 1500-year-old Salt Man 2 found in the Chehrabad salt mine in Zanjan province, northwestern Iran. Our study followed established ancient DNA (aDNA) best practices, including: using dedicated, physically separated clean laboratory facilities with strict contamination control procedures, such as UV sterilization; (ii) independently amplifying multiple short overlapping fragments (101–118 bp), consistent with the expected molecular fragmentation of ancient samples; (iii) confirming amplification success across multiple independent PCR reactions; including three negative controls without nits in each extraction batch to check for external contamination; and failing to amplify longer fragments with standard barcode primers, unlike the successful amplification of modern lice samples under the same lab conditions. The selective amplification of only short fragments from the ancient specimens, along with the limited overall success rate (2 out of 9 specimens), is consistent with the highly degraded DNA typical of ancient material. In contrast, modern lice produced full-length barcodes using standard primers in a single reaction, further supporting their authenticity and ruling out lab contamination.

This study identified clade A in head lice nits from Salt Man 2. This clade is the most widespread lineage, having been found in lice from 49 countries across all five continents [25,33]. It has also been identified in ancient head lice nits recovered from shrunken human heads (tsantsas) of mummies from the Shuar/Jivaro tribe in Ecuador, South America [38]. Clade A expanded around 100,000 years ago, coinciding with the out-of-Africa migration of H. sapiens and supporting a co-evolutionary relationship between lice and humans [2,33].

Our analysis of contemporary lice from Gilan and Kurdistan revealed clades A and B, respectively, consistent with previous reports from northern and northwestern Iran [39]. A recent study in northern Iran identified both clades among schoolchildren by sequencing the *cytb* gene and reported high haplotype and nucleotide diversity [40]. The ancient lice from Zanjan exhibited unique haplotypes within clade A, distinct from those previously documented in Iran or elsewhere. Moreover, these ancient haplotypes showed greater genetic divergence from clade A members than did the contemporary nits analyzed in this study.

Iran’s geographic location has long served as a critical corridor for human migration, linking Africa, Europe, and Asia. In more recent history, trade routes such as the Silk Road likely facilitated human movement and, consequently, the dispersal of human lice. While this study offers initial insights into the ancient genetic diversity of lice in Iran, further investigations using a more comprehensive dataset, including additional museum and contemporary specimens, are needed. Ancient head-louse nit cement has recently been shown to preserve human, pathogen, environmental, and louse DNA, providing a valuable non-destructive resource for paleogenomic studies [41].

These findings provide preliminary insights into the genetic diversity of ancient human lice in Iran. Future studies incorporating larger numbers of ancient and contemporary specimens from diverse geographic regions will be needed to determine whether the haplotypes identified in Salt Man 2 represent extinct lineages or persist in present-day populations. Such investigations may further improve our understanding of the evolutionary history of human lice and their association with human migrations.

## Figures and Tables

**Table 1:** Data on the primers used for amplification and sequencing of the 658-bp COI barcode region from ancient and recent nits. *, Size of amplicons excluding the primers.

Fragment	Primer	Sequence 5′-3′	Start	End	Size *	Reference
F1	C_LepFolF PHdegR1	RKTCAACMAATCATAAAGATATTGG GCGTGAGAAGTAACAAATAC	1	118	118	[22]
F2	PHdegF2 PHdegR2	AATCCGGTTAGAACTTTCTAG TTGAAGGAACYARTCAATTWGC	82	187	106	This study
F3	PHdegF3 PHdegR3	TGATTTTTTTTATAGTTATGCCTG GAACAAATGAGCTACTAATAAG	171	286	116	This study
F4	PHdegF4 PHdegR4	ATAAATAACATRAGYTATTGACTT TAAATCAACTGAAACAGAAGG	267	367	101	This study
F5	PHdegF5 PHdegR5	CTGTTTATCCTCCTCTYAGG CGAACTAARCCAAAATATTKAGG	353	469	117	This study
F6	PHdegF6 PHdegR6	AGGATCAGTAAATTTTATTAGAAC GCATTGTAATAGCTCCAGC	454	559	106	This study
F7	PHdegF7 PHdegR7	GTTGGTAACAGCCTTTTTAYTATT TAAACTTCWGGRTGWCCAAAAAATCA	541	658	118	This study
Short	Phdeg_shortF1 Phdeg_shortR1	TTATTAGTAGCTCATTTGTTC CCTTCTGTTTCAGTTGATTTA	309	367	59	This study

**Table 2:** Data on ancient and recent nits and generated sequences used in this study.

No.	Province/City	Date of Collection	Sample ID	Amplicon Size	GenBank Acc. No.
1	Zanjan/Chehrabad *	2017	MLICE004-18	557	N/A
2			MLICE005-18	658	PV427006
3			MLICE007-18	478	PV427007
4	Gilan/Rasht	2017	L-N-1	658	PV426995
5			L-N-2	658	PV426996
6			L-N-3	658	PV426997
7			L-N-4	658	PV426998
8			L-N-5	610	PV426999
9			L-N-6	658	PV427000
10	Kurdistan/Sanandaj		L-W-1	0	N/A
11			L-W-2	658	PV427001
12			L-W-3	658	PV427002
13			L-W-4	0	N/A
14			L-W-5	658	PV427003
15			L-W-6	658	PV427004
16			L-W-7	557	PV427005

*, Ancient lice.

## Data Availability

The sequence data have been deposited in GenBank under accession numbers PV426995–PV427007.

## Supplementary Materials

The following supporting information can be downloaded at: https://www.mdpi.com/article/10.3390/insects17090929/s1, Supplementary data S1: Sanger sequencing chromatograms of head-louse samples; Supplementary data S2: PCR amplification of ancient head lice nits using overlapping primer sets and a mini-barcode assay; Supplementary data S3: Alignment of COXI sequences generated from ancient lice; Supplementary data S4: BOLD TaxonID Tree.
