# Supplementary material for: DNA Barcoding of Head Lice from a ~1500-Year-Old Salt-Preserved Mummy from the Chehrabad Salt Mine, Iran
Source: Insects. 2026 Sep 4;17(9):929. doi: 10.3390/insects17090929 (PMC13607110; doi:10.3390/insects17090929)
Supplement: Supplementary file 1 [file insects-17-00929-s001.zip › Supplementary data S2 PCR amplification of ancient head lice nits using overlapping primer sets and a mini-barcode assay.pdf]

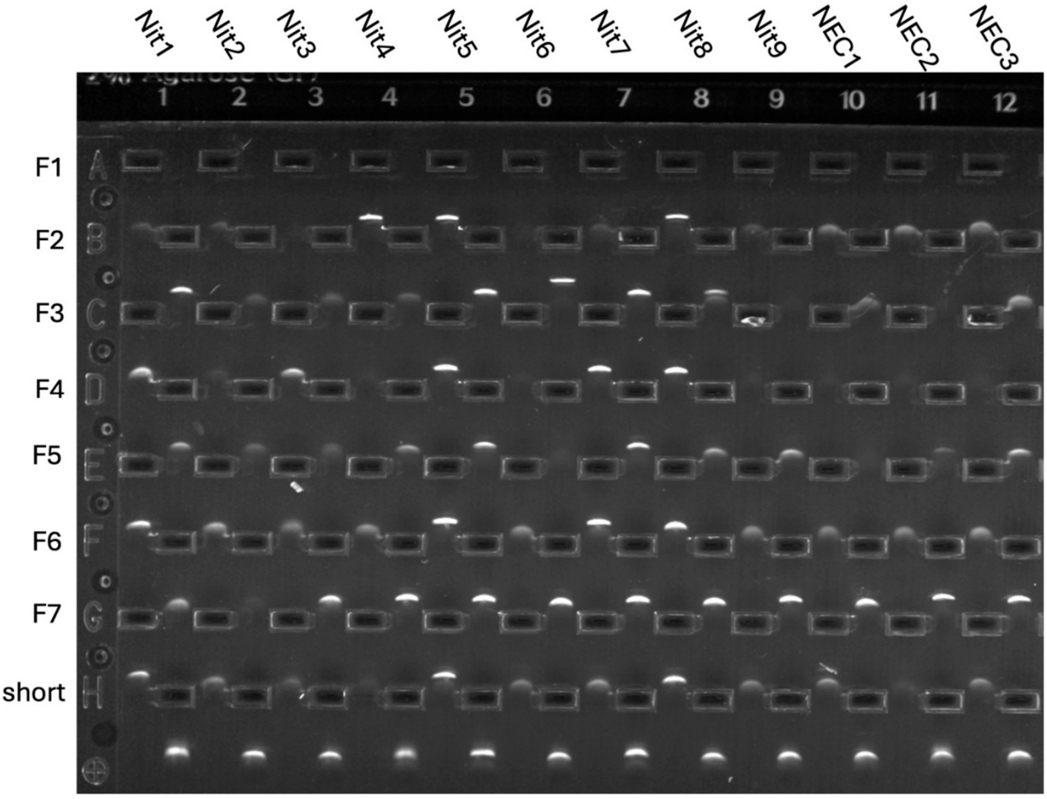

PCR amplification of ancient head lice nits using overlapping primer sets and a mini-barcode assay. A 2% agarose gel showing PCR amplification results. Columns 1–9 show ancient nits (Nit1–Nit9), and columns 10–12 show negative extraction controls (NEC1–NEC3). Rows F1–F7 correspond to overlapping primer sets, and the Short row corresponds to the mini-barcode assay. Bands at the bottom of the gel represent residual primers and primer dimers, commonly observed in high-throughput PCR assays.

supplementarysupplementary
