# Supplementary material for: DNA Barcoding of Head Lice from a ~1500-Year-Old Salt-Preserved Mummy from the Chehrabad Salt Mine, Iran
Source: Insects. 2026 Sep 4;17(9):929. doi: 10.3390/insects17090929 (PMC13607110; doi:10.3390/insects17090929)
Supplement: Supplementary file 1 [file insects-17-00929-s001.zip › Supplementary data S3 Alignment of COXI sequences generated from ancient lice.pdf]

```
          ....|....| ....|....| ....|....| ....|....| ....|....| ....|....|
                  10         20         30         40         50         60
MLICE005  ATTTTTATAC TTATGCTCTG GAGTTTGGTT TGGACTTTTA GGCTTAAGGT TAAGGTTAAT
MLICE004  ATTTTTATAC TTATGCTCTG GAGTTTGGTT TGGACTTTTA GGCTTAAGGT TATGGTTAAT
MLICE007  ---------- ---------- ---------- ---------- ---------- ----------

          ....|....| ....|....| ....|....| ....|....| ....|....| ....|....|
                  70         80         90        100        110        120
MLICE005  AATCCGGTTA GAACTTTCTA GAACAGGCTT GCTTTTGTCC GATAGACACC TATATAACGT
MLICE004  AATCCGGTTA GAACTTTCTA GAACAGGCTT GCTTTTGTCT GATAGATACT TGTATAACNN
MLICE007  ---------- ---------- -AACAGGCTT GCTTTTGTCT GATAGACACC TATATAACGT

          ....|....| ....|....| ....|....| ....|....| ....|....| ....|....|
                 130        140        150        160        170        180
MLICE005  ATTTGTTACT TCTCACGCTT TTGTAATGAT TTTTTTTATA GTTATGCCTG TAATAATAGG
MLICE004  NNNNNNNNNN NNNNNNNNNN NNNNNNNNNN NNNNNNNNNN NNNNNNNNNN NNNNNNNNNN
MLICE007  ACTTGTTACT TCTCACGCTT TTGTAATGAT TTTTTTTATA GTTATGCCTG TAATAATAGG

          ....|....| ....|....| ....|....| ....|....| ....|....| ....|....|
                 190        200        210        220        230        240
MLICE005  CGGTTTTGCA AATTGATTAG TTCCTTCAAT ATTAGGGTCT CCAGATATAG CATTTCCTCG
MLICE004  NNNNNNNNNN NNNNNNNNNN NNNNNNNNNN NNNNNNNNNN NNNNNNNNNN NNNNNNNNNN
MLICE007  CGGTTTTGCA AATTGACTAG TTCCTTCAAT ATTAGGGTCT CCAGATATAG CATTTCCTCG

          ....|....| ....|....| ....|....| ....|....| ....|....| ....|....|
                 250        260        270        280        290        300
MLICE005  TATAAATAAT ATGAGTTATT GACTTCTCAC ACCCTCTGGG ATTTTGCTTA TTAGTAGCTC
MLICE004  NNNNNNNNNN NNNNNNNNNN NNNNNNNNNN NNNNNNNNNN NNNNNNNNNN NNNNNNNNNN
MLICE007  TATAAATAAT ATGAGTTATT GACTTCTCAC ACCCTCTGGG ATTTTGCTTA TTAGTAGCTC

          ....|....| ....|....| ....|....| ....|....| ....|....| ....|....|
                 310        320        330        340        350        360
MLICE005  ATTTGTTCAA GGTGGTGTGG GTACTGGTTG GACTGTTTAT CCCCCTCTTA GGTCTCTAGA
MLICE004  NNNNNNNNAA GGTGGTGTTG GTACTGGCTG GACTGTTTAT CCCCCTCTTA GGTCTCTAGA
MLICE007  GTTTGTTCAA GGCGGTGTTG GTACTGGCTG GACTGTTTAT CCCCCTCTTA GGTCTCTAGA

          ....|....| ....|....| ....|....| ....|....| ....|....| ....|....|
                 370        380        390        400        410        420
MLICE005  AGGCCAACCT TCTGTTTCAG TTGATTTAGC TATTTTAAGT CTTCATTTAG CAGGAGTGAG
MLICE004  AGGNCAANNN NNNNNNNNNN NNNNNNNNNN NNNNNNNNNN NNNNNNNNNN NNNNNNNNNN
MLICE007  AGGCCAACCT TCTGTTTCAG TTGATTTAGC TATTTTAAGT CTTCATTTAG CAGGAGTGAG

          ....|....| ....|....| ....|....| ....|....| ....|....| ....|....|
                 430        440        450        460        470        480
MLICE005  TTCGATTTTA GGATCAGTAA ATTTTATTAG AACTATTTTT AACATATGGC CTCAATATTT
MLICE004  NNNNNNNNNN NNNNNNNNNN NNNNNNNNNN NNNTATTTTT AACATATGGC CTCAATATTT
MLICE007  TTCGATTTTA GGATCAGTAA ATTTTATTAG AACTATTTTT AACATATGGC CTCAATATTT

          ....|....| ....|....| ....|....| ....|....| ....|....| ....|....|
```

```
                490       500       510       520       530       540
MLICE005   TGGCTTAGTT CGACTGCCTT TATTTTGCTG GAGAGTGTTG GTAACAGCCT TTTTATTATT
MLICE004   TGGCTTAGTT CGACTGCCTT TATTTTGCTG GAGAGTGTTG GTAACAGCCT TTTTGTTATT
MLICE007   TGGCTTAGTT CGACTGCCTT TATTTTGCTG GAGAGTGTTG GTAACAGCCT TTTTGTTATT

           ....|....| ....|....| ....|....| ....|....| ....|....| ....|....|
                550       560       570       580       590       600
MLICE005   ACTGTCACTT CCAGTTTTAG CTGGAGCTAT TACAATGCTC TTAATAGACC GTAATTTCAA
MLICE004   ACTGTCACTT CCAGTTTTA- ---------- ---------- ---------- ----------
MLICE007   ACTGTCACTT CCAGTTTTA- ---------- ---------- ---------- ----------

           ....|....| ....|....| ....|....| ....|....| ....|....| ....|...
                610       620       630       640       650
MLICE005   TTGCTCATTT TTTGATCCTT TAGGGGGTGG TGATCCTGTT TTATACCAAC ATTTATTT
MLICE004   ---------- ---------- ---------- ---------- ---------- --------
MLICE007   ---------- ---------- ---------- ---------- ---------- --------
```

Supplementary data 3: Alignment of COXI sequences generated from ancient lice.
