# Supplementary material for: DNA Barcoding of Head Lice from a ~1500-Year-Old Salt-Preserved Mummy from the Chehrabad Salt Mine, Iran
Source: Insects. 2026 Sep 4;17(9):929. doi: 10.3390/insects17090929 (PMC13607110; doi:10.3390/insects17090929)
Supplement: Supplementary file 1 [file insects-17-00929-s001.zip › Supplementary data S4 BOLD TaxonID Tree.pdf]

BOLD TaxonID Tree

Title : Tree Result - Search: Marker(COI-5P); Tax(Pediculus humanus; Pediculus humanus capitis); Include public records (770 records returned) (770 records selected)

Date : 20-Feb-2023

Data Type : Nucleotide

Distance Model : Kimura 2 Parameter

Marker : COI-5P

Colourization : [blue]=Stop Codons [red]=Contamination or misidentification

Label : Process ID

Label : Taxon

Label : Extra Info

Label : Country

Label : Barcode Cluster (BIN)

Filter : length > 200bp only

Filter : exclude records flagged as misidentifications

Filter : exclude records with stop codons

Filter : exclude contaminants

Sequence Count : 760

Species count : 1

Genus count : 1

Family count : 1

Unidentified : 0

BIN Count : 4

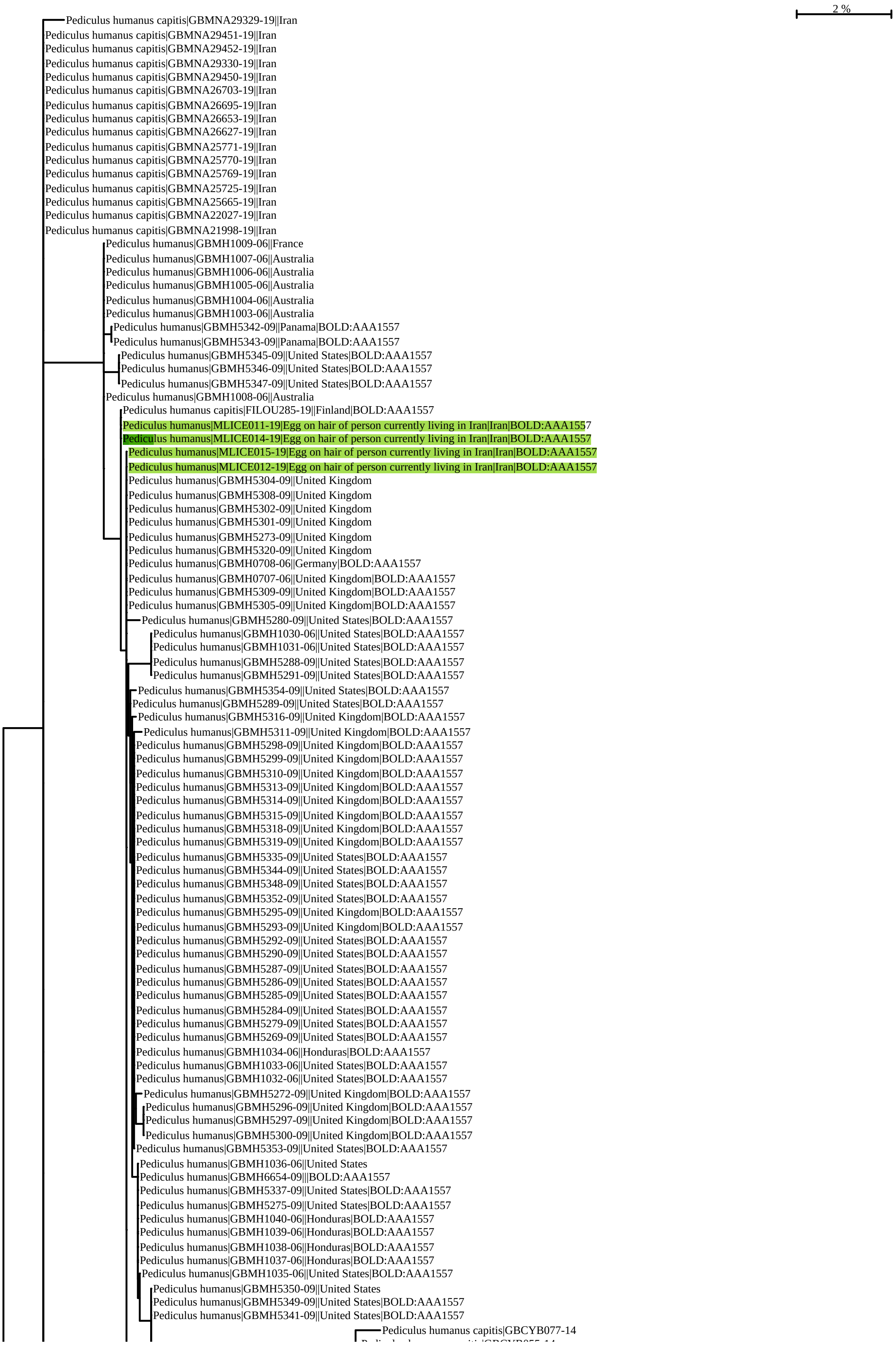
2 %
Pediculus humanus capitis|GBMNA29329-19||Iran
Pediculus humanus capitis|GBMNA29451-19||Iran
Pediculus humanus capitis|GBMNA29452-19||Iran
Pediculus humanus capitis|GBMNA29330-19||Iran
Pediculus humanus capitis|GBMNA29450-19||Iran
Pediculus humanus capitis|GBMNA26703-19||Iran
Pediculus humanus capitis|GBMNA26695-19||Iran
Pediculus humanus capitis|GBMNA26653-19||Iran
Pediculus humanus capitis|GBMNA26627-19||Iran
Pediculus humanus capitis|GBMNA25771-19||Iran
Pediculus humanus capitis|GBMNA25770-19||Iran
Pediculus humanus capitis|GBMNA25769-19||Iran
Pediculus humanus capitis|GBMNA25725-19||Iran
Pediculus humanus capitis|GBMNA25665-19||Iran
Pediculus humanus capitis|GBMNA22027-19||Iran
Pediculus humanus capitis|GBMNA21998-19||Iran
Pediculus humanus|GBMH1009-06||France
Pediculus humanus|GBMH1007-06||Australia
Pediculus humanus|GBMH1006-06||Australia
Pediculus humanus|GBMH1005-06||Australia
Pediculus humanus|GBMH1004-06||Australia
Pediculus humanus|GBMH1003-06||Australia
Pediculus humanus|GBMH5342-09||Panama|BOLD:AAA1557
Pediculus humanus|GBMH5343-09||Panama|BOLD:AAA1557
Pediculus humanus|GBMH5345-09||United States|BOLD:AAA1557
Pediculus humanus|GBMH5346-09||United States|BOLD:AAA1557
Pediculus humanus|GBMH5347-09||United States|BOLD:AAA1557
Pediculus humanus|GBMH1008-06||Australia
Pediculus humanus capitis|FILOU285-19||Finland|BOLD:AAA1557
Pediculus humanus|MLICE011-19|Egg on hair of person currently living in Iran|Iran|BOLD:AAA1557
Pediculus humanus|MLICE014-19|Egg on hair of person currently living in Iran|Iran|BOLD:AAA1557
Pediculus humanus|MLICE015-19|Egg on hair of person currently living in Iran|Iran|BOLD:AAA1557
Pediculus humanus|MLICE012-19|Egg on hair of person currently living in Iran|Iran|BOLD:AAA1557
Pediculus humanus|GBMH5304-09||United Kingdom
Pediculus humanus|GBMH5308-09||United Kingdom
Pediculus humanus|GBMH5302-09||United Kingdom
Pediculus humanus|GBMH5301-09||United Kingdom
Pediculus humanus|GBMH5273-09||United Kingdom
Pediculus humanus|GBMH5320-09||United Kingdom
Pediculus humanus|GBMH0708-06||Germany|BOLD:AAA1557
Pediculus humanus|GBMH0707-06||United Kingdom|BOLD:AAA1557
Pediculus humanus|GBMH5309-09||United Kingdom|BOLD:AAA1557
Pediculus humanus|GBMH5305-09||United Kingdom|BOLD:AAA1557
Pediculus humanus|GBMH5280-09||United States|BOLD:AAA1557
Pediculus humanus|GBMH1030-06||United States|BOLD:AAA1557
Pediculus humanus|GBMH1031-06||United States|BOLD:AAA1557
Pediculus humanus|GBMH5288-09||United States|BOLD:AAA1557
Pediculus humanus|GBMH5291-09||United States|BOLD:AAA1557
Pediculus humanus|GBMH5354-09||United States|BOLD:AAA1557
Pediculus humanus|GBMH5289-09||United States|BOLD:AAA1557
Pediculus humanus|GBMH5316-09||United Kingdom|BOLD:AAA1557
Pediculus humanus|GBMH5311-09||United Kingdom|BOLD:AAA1557
Pediculus humanus|GBMH5298-09||United Kingdom|BOLD:AAA1557
Pediculus humanus|GBMH5299-09||United Kingdom|BOLD:AAA1557
Pediculus humanus|GBMH5310-09||United Kingdom|BOLD:AAA1557
Pediculus humanus|GBMH5313-09||United Kingdom|BOLD:AAA1557
Pediculus humanus|GBMH5314-09||United Kingdom|BOLD:AAA1557
Pediculus humanus|GBMH5315-09||United Kingdom|BOLD:AAA1557
Pediculus humanus|GBMH5318-09||United Kingdom|BOLD:AAA1557
Pediculus humanus|GBMH5319-09||United Kingdom|BOLD:AAA1557
Pediculus humanus|GBMH5335-09||United States|BOLD:AAA1557
Pediculus humanus|GBMH5344-09||United States|BOLD:AAA1557
Pediculus humanus|GBMH5348-09||United States|BOLD:AAA1557
Pediculus humanus|GBMH5352-09||United States|BOLD:AAA1557
Pediculus humanus|GBMH5295-09||United Kingdom|BOLD:AAA1557
Pediculus humanus|GBMH5293-09||United Kingdom|BOLD:AAA1557
Pediculus humanus|GBMH5292-09||United States|BOLD:AAA1557
Pediculus humanus|GBMH5290-09||United States|BOLD:AAA1557
Pediculus humanus|GBMH5287-09||United States|BOLD:AAA1557
Pediculus humanus|GBMH5286-09||United States|BOLD:AAA1557
Pediculus humanus|GBMH5285-09||United States|BOLD:AAA1557
Pediculus humanus|GBMH5284-09||United States|BOLD:AAA1557
Pediculus humanus|GBMH5279-09||United States|BOLD:AAA1557
Pediculus humanus|GBMH5269-09||United States|BOLD:AAA1557
Pediculus humanus|GBMH1034-06||Honduras|BOLD:AAA1557
Pediculus humanus|GBMH1033-06||United States|BOLD:AAA1557
Pediculus humanus|GBMH1032-06||United States|BOLD:AAA1557
Pediculus humanus|GBMH5272-09||United Kingdom|BOLD:AAA1557
Pediculus humanus|GBMH5296-09||United Kingdom|BOLD:AAA1557
Pediculus humanus|GBMH5297-09||United Kingdom|BOLD:AAA1557
Pediculus humanus|GBMH5300-09||United Kingdom|BOLD:AAA1557
Pediculus humanus|GBMH5353-09||United States|BOLD:AAA1557
Pediculus humanus|GBMH1036-06||United States
Pediculus humanus|GBMH6654-09|||BOLD:AAA1557
Pediculus humanus|GBMH5337-09||United States|BOLD:AAA1557
Pediculus humanus|GBMH5275-09||United States|BOLD:AAA1557
Pediculus humanus|GBMH1040-06||Honduras|BOLD:AAA1557
Pediculus humanus|GBMH1039-06||Honduras|BOLD:AAA1557
Pediculus humanus|GBMH1038-06||Honduras|BOLD:AAA1557
Pediculus humanus|GBMH1037-06||Honduras|BOLD:AAA1557
Pediculus humanus|GBMH1035-06||United States|BOLD:AAA1557
Pediculus humanus|GBMH5350-09||United States
Pediculus humanus|GBMH5349-09||United States|BOLD:AAA1557
Pediculus humanus|GBMH5341-09||United States|BOLD:AAA1557
Pediculus humanus capitis|GBCYB077-14

Pediculus humanus|GBMH5349-09||United States|BOLD:AAA1557
Pediculus humanus|GBMH5341-09||United States|BOLD:AAA1557
Pediculus humanus capitis|GBCYB077-14
Pediculus humanus capitis|GBCYB055-14
Pediculus humanus capitis|GBCYB074-14
Pediculus humanus capitis|GBCYB075-14
Pediculus humanus capitis|GBCYB076-14
Pediculus humanus capitis|GBCYB035-14
Pediculus humanus capitis|GBCYB046-14
Pediculus humanus capitis|GBCYB079-14
Pediculus humanus capitis|GBCYB036-14||Peru
Pediculus humanus capitis|GBCYB049-14
Pediculus humanus capitis|GBCYB050-14
Pediculus humanus capitis|GBCYB051-14
Pediculus humanus capitis|GBCYB078-14
Pediculus humanus capitis|GBCYB080-14
Pediculus humanus capitis|GBCYB054-14
Pediculus humanus capitis|GBCYB071-14
Pediculus humanus capitis|GBCYB063-14
Pediculus humanus capitis|GBCYB053-14
Pediculus humanus capitis|GBCYB062-14
Pediculus humanus capitis|GBCYB066-14
Pediculus humanus capitis|GBCYB048-14
Pediculus humanus capitis|GBCYB057-14
Pediculus humanus capitis|GBCYB068-14
Pediculus humanus|GBCYB081-14
Pediculus humanus capitis|GBCYB073-14
Pediculus humanus capitis|GBCYB072-14
Pediculus humanus capitis|GBCYB070-14
Pediculus humanus capitis|GBCYB069-14
Pediculus humanus capitis|GBCYB067-14
Pediculus humanus capitis|GBCYB065-14
Pediculus humanus capitis|GBCYB061-14
Pediculus humanus capitis|GBCYB060-14
Pediculus humanus capitis|GBCYB059-14
Pediculus humanus capitis|GBCYB058-14
Pediculus humanus capitis|GBCYB056-14
Pediculus humanus capitis|GBCYB052-14
Pediculus humanus capitis|GBCYB047-14
Pediculus humanus capitis|GBCYB045-14
Pediculus humanus capitis|GBCYB044-14
Pediculus humanus capitis|GBCYB034-14
Pediculus humanus capitis|GBCYB009-14||Turkey
Pediculus humanus capitis|GBCYB008-14||Turkey
Pediculus humanus capitis|GBCYB064-14
Pediculus humanus capitis|GBCYB082-14||United Kingdom
Pediculus humanus capitis|GBCYB083-14||Netherlands
Pediculus humanus|GBMH5312-09||United Kingdom
Pediculus humanus|MLICE016-19|Egg on hair of person currently living in Iran|Iran|BOLD:AAA1557
Pediculus humanus capitis|GBMNA27281-19||Iran
Pediculus humanus capitis|GBMNA27293-19||Iran
Pediculus humanus capitis|GBMNA29305-19||Iran
Pediculus humanus capitis|GBMNA29306-19||Iran
Pediculus humanus capitis|GBMNA30478-19||Iran
Pediculus humanus capitis|GBMNA30479-19||Iran
Pediculus humanus capitis|GBMNA31113-19||Iran
Pediculus humanus capitis|GBMNA31224-19||Iran
Pediculus humanus capitis|GBMNA31226-19||Iran
Pediculus humanus|GBMH0625-06||Portugal|BOLD:AAA1557
Pediculus humanus capitis|GBMNB60231-20||Pakistan|BOLD:AAA1556
Pediculus humanus capitis|GBMNB60229-20||Pakistan|BOLD:AAA1556
Pediculus humanus|GBMH1015-06||Nepal
Pediculus humanus|GBMH1014-06||Nepal
Pediculus humanus|GBMH1013-06||Nepal
Pediculus humanus|GBMH0981-06||Iran
Pediculus humanus|GBMH0980-06||Iran
Pediculus humanus|GBMH0979-06||Argentina
Pediculus humanus|GBMH0978-06||Papua New Guinea
Pediculus humanus|GBMH0977-06||Kenya
Pediculus humanus|GBMH0976-06||Israel
Pediculus humanus|GBMH0975-06||Australia
Pediculus humanus|GBMH0974-06||Nepal
Pediculus humanus|GBMH5332-09||Papua New Guinea|BOLD:AAA1556
Pediculus humanus|GBMH5331-09||Papua New Guinea|BOLD:AAA1556
Pediculus humanus|GBMH5328-09||Colombia|BOLD:AAA1556
Pediculus humanus|GBMH5327-09||Colombia|BOLD:AAA1556
Pediculus humanus|GBMH5326-09||Ecuador|BOLD:AAA1556
Pediculus humanus|GBMH5325-09||Ecuador|BOLD:AAA1556
Pediculus humanus|GBMH5323-09||Mongolia|BOLD:AAA1556
Pediculus humanus|GBMH5322-09||Burundi|BOLD:AAA1556
Pediculus humanus|GBMH5321-09||Burundi|BOLD:AAA1556
Pediculus humanus|GBMH1088-06||Papua New Guinea|BOLD:AAA1556
Pediculus humanus|GBMH1087-06||Papua New Guinea|BOLD:AAA1556
Pediculus humanus|GBMH1086-06||Papua New Guinea|BOLD:AAA1556
Pediculus humanus|GBMH1085-06||Papua New Guinea|BOLD:AAA1556
Pediculus humanus|GBMH1084-06||Philippines|BOLD:AAA1556
Pediculus humanus|GBMH1083-06||Philippines|BOLD:AAA1556
Pediculus humanus|GBMH1081-06||Honduras|BOLD:AAA1556
Pediculus humanus|GBMH1074-06||Papua New Guinea|BOLD:AAA1556
Pediculus humanus|GBMH1073-06||Papua New Guinea|BOLD:AAA1556
Pediculus humanus|GBMH1072-06||Papua New Guinea|BOLD:AAA1556
Pediculus humanus|GBMH1067-06||Philippines|BOLD:AAA1556
Pediculus humanus|GBMH1066-06||Honduras|BOLD:AAA1556
Pediculus humanus|GBMH1065-06||Philippines|BOLD:AAA1556
Pediculus humanus|GBMH1064-06||Yemen|BOLD:AAA1556
Pediculus humanus|GBMH1063-06||United States|BOLD:AAA1556
Pediculus humanus|GBMH1062-06||United States|BOLD:AAA1556
Pediculus humanus|GBMH1061-06||United States|BOLD:AAA1556
Pediculus humanus|GBMH1060-06||United States|BOLD:AAA1556

[illegible]
Pediculus humanus|GBMH1062-06||United States|BOLD:AAA1556
Pediculus humanus|GBMH1061-06||United States|BOLD:AAA1556
Pediculus humanus|GBMH1060-06||United States|BOLD:AAA1556
Pediculus humanus|GBMH1059-06||Canada|BOLD:AAA1556
Pediculus humanus|GBMH1058-06||Canada|BOLD:AAA1556
Pediculus humanus|GBMH1057-06||Canada|BOLD:AAA1556
Pediculus humanus|GBMH1056-06||Canada|BOLD:AAA1556
Pediculus humanus|GBMH1055-06||Canada|BOLD:AAA1556
Pediculus humanus|GBMH1054-06||Canada|BOLD:AAA1556
Pediculus humanus|GBMH1052-06||United States|BOLD:AAA1556
Pediculus humanus|GBMH1051-06||United States|BOLD:AAA1556
Pediculus humanus|GBMH1050-06||United States|BOLD:AAA1556
Pediculus humanus|GBMH1049-06||United States|BOLD:AAA1556
Pediculus humanus|GBMH1048-06||United States|BOLD:AAA1556
Pediculus humanus capitis|GBMNB60289-20|||BOLD:AAA1556
Pediculus humanus capitis|GBMNB60288-20|||BOLD:AAA1556
Pediculus humanus capitis|GBMNB60286-20|||BOLD:AAA1556
Pediculus humanus capitis|GBMNB60284-20|||BOLD:AAA1556
Pediculus humanus capitis|GBMNB60283-20|||BOLD:AAA1556
Pediculus humanus capitis|GBMNB60282-20|||BOLD:AAA1556
Pediculus humanus capitis|GBMNB60281-20|||BOLD:AAA1556
Pediculus humanus capitis|GBMNB60280-20|||BOLD:AAA1556
Pediculus humanus capitis|GBMNB60279-20|||BOLD:AAA1556
Pediculus humanus capitis|GBMNB60275-20|||BOLD:AAA1556
Pediculus humanus capitis|GBMNB60272-20|||BOLD:AAA1556
Pediculus humanus capitis|GBMNB60269-20|||BOLD:AAA1556
Pediculus humanus capitis|GBMNB60268-20|||BOLD:AAA1556
Pediculus humanus capitis|GBMNB60267-20|||BOLD:AAA1556
Pediculus humanus capitis|GBMNB60264-20|||BOLD:AAA1556
Pediculus humanus capitis|GBMNB60263-20|||BOLD:AAA1556
Pediculus humanus capitis|GBMNB60262-20|||BOLD:AAA1556
Pediculus humanus capitis|GBMNB60260-20|||BOLD:AAA1556
Pediculus humanus capitis|GBMNB60258-20|||BOLD:AAA1556
Pediculus humanus capitis|GBMNB60257-20|||BOLD:AAA1556
Pediculus humanus capitis|GBMNB60251-20|||BOLD:AAA1556
Pediculus humanus capitis|GBMNB60248-20|||BOLD:AAA1556
Pediculus humanus capitis|GBMNB60247-20|||BOLD:AAA1556
Pediculus humanus capitis|GBMNB60246-20|||BOLD:AAA1556
Pediculus humanus capitis|GBMNB60243-20|||BOLD:AAA1556
Pediculus humanus capitis|GBMNB60240-20|||BOLD:AAA1556
Pediculus humanus capitis|GBMNB60239-20|||BOLD:AAA1556
Pediculus humanus capitis|GBMNB60238-20|||BOLD:AAA1556
Pediculus humanus capitis|GBMNB60237-20|||BOLD:AAA1556
Pediculus humanus capitis|GBMNB60236-20|||BOLD:AAA1556
Pediculus humanus capitis|GBMNB60235-20|||BOLD:AAA1556
Pediculus humanus capitis|GBMNB60234-20|||BOLD:AAA1556
Pediculus humanus capitis|GBMNB60233-20|||BOLD:AAA1556
Pediculus humanus capitis|GBMNB60232-20|||BOLD:AAA1556
Pediculus humanus capitis|GBMHP361-15|taxon:121226|Thailand|BOLD:AAA1556
Pediculus humanus capitis|GBMHP360-15|taxon:121226|Thailand|BOLD:AAA1556
Pediculus humanus capitis|GBMHP359-15|taxon:121226|Thailand|BOLD:AAA1556
Pediculus humanus capitis|GBMHP358-15|taxon:121226|Thailand|BOLD:AAA1556
Pediculus humanus capitis|GBMHP350-15|taxon:121226|Thailand|BOLD:AAA1556
Pediculus humanus capitis|GBMHP349-15|taxon:121226|Thailand|BOLD:AAA1556
Pediculus humanus capitis|GBMHP344-15|taxon:121226|Thailand|BOLD:AAA1556
Pediculus humanus capitis|GBMHP343-15|taxon:121226|Thailand|BOLD:AAA1556
Pediculus humanus capitis|GBMHP342-15|taxon:121226|Thailand|BOLD:AAA1556
Pediculus humanus capitis|GBMHP341-15|taxon:121226|Thailand|BOLD:AAA1556
Pediculus humanus capitis|GBMHP340-15|taxon:121226|Thailand|BOLD:AAA1556
Pediculus humanus capitis|GBMHP338-15|taxon:121226|Thailand|BOLD:AAA1556
Pediculus humanus capitis|GBMHP336-15|taxon:121226|Thailand|BOLD:AAA1556
Pediculus humanus capitis|GBMHP335-15|taxon:121226|Thailand|BOLD:AAA1556
Pediculus humanus capitis|GBMHP334-15|taxon:121226|Thailand|BOLD:AAA1556
Pediculus humanus capitis|GBMHP332-15|taxon:121226|Thailand|BOLD:AAA1556
Pediculus humanus capitis|GBMHP324-15|taxon:121226|Thailand|BOLD:AAA1556
Pediculus humanus capitis|GBMHP320-15|taxon:121226|Thailand|BOLD:AAA1556
Pediculus humanus capitis|GBMHP315-15|taxon:121226|Thailand|BOLD:AAA1556
Pediculus humanus capitis|GBMHP291-15|taxon:121226|Thailand|BOLD:AAA1556
Pediculus humanus capitis|GBMHP286-15|taxon:121226|Thailand|BOLD:AAA1556
Pediculus humanus capitis|GBMHP284-15|taxon:121226|Thailand|BOLD:AAA1556
Pediculus humanus capitis|GBMHP260-15|taxon:121226|Thailand|BOLD:AAA1556
Pediculus humanus capitis|GBMHP258-15|taxon:121226|Thailand|BOLD:AAA1556
Pediculus humanus capitis|GBMHP257-15|taxon:121226|Thailand|BOLD:AAA1556
Pediculus humanus capitis|GBMHP255-15|taxon:121226|Thailand|BOLD:AAA1556
Pediculus humanus capitis|GBMHP250-15|taxon:121226|Thailand|BOLD:AAA1556
Pediculus humanus capitis|GBMHP240-15|taxon:121226|Thailand|BOLD:AAA1556
Pediculus humanus capitis|GBMHP239-15|taxon:121226|Thailand|BOLD:AAA1556
Pediculus humanus capitis|GBMHP238-15|taxon:121226|Thailand|BOLD:AAA1556
Pediculus humanus capitis|GBMHP237-15|taxon:121226|Thailand|BOLD:AAA1556
Pediculus humanus capitis|GBMHP236-15|taxon:121226|Thailand|BOLD:AAA1556
Pediculus humanus capitis|GBMHP234-15|taxon:121226|Thailand|BOLD:AAA1556
Pediculus humanus capitis|GBMHP229-15|taxon:121226|Thailand|BOLD:AAA1556
Pediculus humanus capitis|GBMHP224-15|taxon:121226|Thailand|BOLD:AAA1556
Pediculus humanus capitis|GBMHP209-15|taxon:121226|Thailand|BOLD:AAA1556
Pediculus humanus capitis|GBMHP207-15|taxon:121226|Thailand|BOLD:AAA1556
Pediculus humanus capitis|GBMHP201-15|taxon:121226|Thailand|BOLD:AAA1556
Pediculus humanus capitis|GBMHP200-15|taxon:121226|Thailand|BOLD:AAA1556
Pediculus humanus capitis|GBMHP198-15|taxon:121226|Thailand|BOLD:AAA1556
Pediculus humanus capitis|GBMHP195-15|taxon:121226|Thailand|BOLD:AAA1556
Pediculus humanus capitis|GBMHP192-15|taxon:121226|Thailand|BOLD:AAA1556
Pediculus humanus capitis|GBMHP185-15|taxon:121226|Thailand|BOLD:AAA1556
Pediculus humanus capitis|GBMHP181-15|taxon:121226|Thailand|BOLD:AAA1556
Pediculus humanus capitis|GBMHP178-15|taxon:121226|Thailand|BOLD:AAA1556
Pediculus humanus capitis|GBMHP175-15|taxon:121226|Thailand|BOLD:AAA1556
Pediculus humanus|GBMH0623-06||Russia|BOLD:AAA1556
Pediculus humanus|GBMH0705-06||United States|BOLD:AAA1556
Pediculus humanus|GBMH0706-06||Ecuador|BOLD:AAA1556
Pediculus humanus capitis|GBMHP174-15|taxon:121226|Thailand|BOLD:AAA1556

Pediculus humanus|GBMH0705-06||United States|BOLD:AAA1556
Pediculus humanus|GBMH0706-06||Ecuador|BOLD:AAA1556
Pediculus humanus capitis|GBMHP174-15|taxon:121226|Thailand|BOLD:AAA1556
Pediculus humanus capitis|GBMHP141-15|taxon:121226|Thailand|BOLD:AAA1556
Pediculus humanus capitis|GBMHP139-15|taxon:121226|Thailand|BOLD:AAA1556
Pediculus humanus capitis|GBMHP138-15|taxon:121226|Thailand|BOLD:AAA1556
Pediculus humanus capitis|GBMHP137-15|taxon:121226|Thailand|BOLD:AAA1556
Pediculus humanus capitis|GBMHP136-15|taxon:121226|Thailand|BOLD:AAA1556
Pediculus humanus capitis|GBMHP134-15|taxon:121226|Thailand|BOLD:AAA1556
Pediculus humanus capitis|GBMHP133-15|taxon:121226|Thailand|BOLD:AAA1556
Pediculus humanus capitis|GBMHP132-15|taxon:121226|Thailand|BOLD:AAA1556
Pediculus humanus capitis|GBMHP130-15|taxon:121226|Thailand|BOLD:AAA1556
Pediculus humanus capitis|GBMHP129-15|taxon:121226|Thailand|BOLD:AAA1556
Pediculus humanus capitis|GBMHP128-15|taxon:121226|Thailand|BOLD:AAA1556
Pediculus humanus capitis|GBMHP127-15|taxon:121226|Thailand|BOLD:AAA1556
Pediculus humanus capitis|GBMHP126-15|taxon:121226|Thailand|BOLD:AAA1556
Pediculus humanus capitis|GBMHP125-15|taxon:121226|Thailand|BOLD:AAA1556
Pediculus humanus capitis|GBMHP123-15|taxon:121226|Thailand|BOLD:AAA1556
Pediculus humanus|GBMH0923-06||Nepal
Pediculus humanus|GBMH0924-06||Nepal
Pediculus humanus|GBMH0925-06||Nepal
Pediculus humanus|GBMH0926-06||Nepal
Pediculus humanus|GBMH0927-06||Nepal
Pediculus humanus|GBMH0928-06||Nepal
Pediculus humanus|GBMH0929-06||Nepal
Pediculus humanus|GBMH0930-06||Nepal
Pediculus humanus|GBMH0931-06||Nepal
Pediculus humanus|GBMH0932-06||Nepal
Pediculus humanus|GBMH0933-06||Nepal
Pediculus humanus|GBMH0934-06||Nepal
Pediculus humanus|GBMH0935-06||Nepal
Pediculus humanus|GBMH0936-06||Nepal
Pediculus humanus|GBMH0937-06||Nepal
Pediculus humanus|GBMH0938-06||Nepal
Pediculus humanus|GBMH0939-06||Nepal
Pediculus humanus|GBMH0940-06||Nepal
Pediculus humanus|GBMH0941-06||Nepal
Pediculus humanus|GBMH0942-06||Nepal
Pediculus humanus|GBMH0943-06||Nepal
Pediculus humanus|GBMH0944-06||Nepal
Pediculus humanus|GBMH0961-06||Nepal
Pediculus humanus|GBMH0962-06||Nepal
Pediculus humanus|GBMH0963-06||Nepal
Pediculus humanus capitis|GBMHP109-15|taxon:121226|Thailand|BOLD:AAA1556
Pediculus humanus capitis|GBMHP116-15|taxon:121226|Thailand|BOLD:AAA1556
Pediculus humanus capitis|GBMHP122-15|taxon:121226|Thailand|BOLD:AAA1556
Pediculus humanus|GBMH0622-06||Zimbabwe|BOLD:AAA1556
Pediculus humanus capitis|GBMHP114-15|taxon:121226|Thailand|BOLD:AAA1556
Pediculus humanus capitis|GBMHP118-15|taxon:121226|Thailand|BOLD:AAA1556
Pediculus humanus capitis|GBMHP119-15|taxon:121226|Thailand|BOLD:AAA1556
Pediculus humanus|GBMH1002-06||Israel
Pediculus humanus capitis|GBMHP306-15|taxon:121226|Thailand|BOLD:AAA1556
Pediculus humanus capitis|GBMHP308-15|taxon:121226|Thailand|BOLD:AAA1556
Pediculus humanus|GBMH0997-06||China
Pediculus humanus|GBMH0998-06||China
Pediculus humanus|GBMH0999-06||China
Pediculus humanus|GBMH1000-06||China
Pediculus humanus capitis|GBMHP151-15|taxon:121226|Thailand|BOLD:AAA1556
Pediculus humanus capitis|GBMHP152-15|taxon:121226|Thailand|BOLD:AAA1556
Pediculus humanus capitis|GBMHP168-15|taxon:121226|Thailand|BOLD:AAA1556
Pediculus humanus capitis|GBMHP328-15|taxon:121226|Thailand|BOLD:AAA1556
Pediculus humanus capitis|GBMHP353-15|taxon:121226|Thailand|BOLD:AAA1556
Pediculus humanus capitis|GBMHP355-15|taxon:121226|Thailand|BOLD:AAA1556
Pediculus humanus|ENTJR340-08||Thailand|BOLD:AAA1556
Pediculus humanus|ENTJR343-08||Thailand|BOLD:AAA1556
Pediculus humanus|ENTJR344-08||Thailand|BOLD:AAA1556
Pediculus humanus|ENTJR345-08||Thailand|BOLD:AAA1556
Pediculus humanus|ENTJR347-08||Thailand|BOLD:AAA1556
Pediculus humanus|SYC504-14||French Polynesia|BOLD:AAA1556
Pediculus humanus capitis|GBMHP148-15|taxon:121226|Thailand|BOLD:AAA1556
Pediculus humanus capitis|GBMHP149-15|taxon:121226|Thailand|BOLD:AAA1556
Pediculus humanus capitis|GBMHP150-15|taxon:121226|Thailand|BOLD:AAA1556
Pediculus humanus capitis|GBMHP167-15|taxon:121226|Thailand|BOLD:AAA1556
Pediculus humanus capitis|GBMHP326-15|taxon:121226|Thailand|BOLD:AAA1556
Pediculus humanus capitis|GBMHP169-15|taxon:121226|Thailand|BOLD:AAA1556
Pediculus humanus|GBMH1045-06||Philippines|BOLD:AAA1556
Pediculus humanus|GBMH1041-06||Philippines|BOLD:AAA1556
Pediculus humanus|GBMH1042-06||Philippines|BOLD:AAA1556
Pediculus humanus|GBMH1043-06||Philippines|BOLD:AAA1556
Pediculus humanus|GBMH1044-06||Philippines|BOLD:AAA1556
Pediculus humanus|GBMH1046-06||Philippines|BOLD:AAA1556
Pediculus humanus|GBMH1047-06||Philippines|BOLD:AAA1556
Pediculus humanus capitis|GBMHP166-15|taxon:121226|Thailand|BOLD:AAA1556
Pediculus humanus|GBMH1068-06||Philippines|BOLD:AAA1556
Pediculus humanus|GBMH1080-06||Philippines|BOLD:AAA1556
Pediculus humanus|GBMH5324-09||Cook Islands|BOLD:AAA1556
Pediculus humanus capitis|GBMHP329-15|taxon:121226|Thailand|BOLD:AAA1556
Pediculus humanus capitis|GBMHP357-15|taxon:121226|Thailand|BOLD:AAA1556
Pediculus humanus capitis|GBMHP376-15|taxon:121226|Thailand|BOLD:AAA1556
Pediculus humanus capitis|GBMHP318-15|taxon:121226|Thailand|BOLD:AAA1556
Pediculus humanus capitis|GBMHP197-15|taxon:121226|Thailand|BOLD:AAA1556
Pediculus humanus capitis|GBMHP184-15|taxon:121226|Thailand|BOLD:AAA1556
Pediculus humanus capitis|GBMHP146-15|taxon:121226|Thailand|BOLD:AAA1556
Pediculus humanus capitis|GBMHP365-15|taxon:121226|Thailand|BOLD:AAA1556
Pediculus humanus capitis|GBMNB60265-20|||BOLD:AAA1556
Pediculus humanus capitis|GBMNB60266-20|||BOLD:AAA1556
Pediculus humanus capitis|GBMNB60271-20|||BOLD:AAA1556
Pediculus humanus capitis|GBMHP363-15|taxon:121226|Thailand

Pediculus humanus capitis|GBMNB60266-20|||BOLD:AAA1556
Pediculus humanus capitis|GBMNB60271-20|||BOLD:AAA1556
Pediculus humanus capitis|GBMHP363-15|taxon:121226|Thailand
Pediculus humanus capitis|GBMHP364-15|taxon:121226|Thailand
Pediculus humanus|GBMIN90430-17||Iran|BOLD:AAA1556
Pediculus humanus capitis|GBMNE24059-21|||BOLD:AAA1556
Pediculus humanus capitis|GBMHP367-15|taxon:121226|Thailand|BOLD:AAA1556
Pediculus humanus capitis|GBMHP317-15|taxon:121226|Thailand|BOLD:AAA1556
Pediculus humanus capitis|GBMHP312-15|taxon:121226|Thailand|BOLD:AAA1556
Pediculus humanus capitis|GBMHP280-15|taxon:121226|Thailand|BOLD:AAA1556
Pediculus humanus capitis|GBMHP279-15|taxon:121226|Thailand|BOLD:AAA1556
Pediculus humanus capitis|GBMHP278-15|taxon:121226|Thailand|BOLD:AAA1556
Pediculus humanus capitis|GBMHP277-15|taxon:121226|Thailand|BOLD:AAA1556
Pediculus humanus capitis|GBMHP205-15|taxon:121226|Thailand|BOLD:AAA1556
Pediculus humanus capitis|GBMHP204-15|taxon:121226|Thailand|BOLD:AAA1556
Pediculus humanus capitis|GBMHP203-15|taxon:121226|Thailand|BOLD:AAA1556
Pediculus humanus capitis|GBMHP202-15|taxon:121226|Thailand|BOLD:AAA1556
Pediculus humanus capitis|GBMHP327-15|taxon:121226|Thailand|BOLD:AAA1556
Pediculus humanus capitis|GBMHP194-15|taxon:121226|Thailand|BOLD:AAA1556
Pediculus humanus capitis|GBMHP193-15|taxon:121226|Thailand|BOLD:AAA1556
Pediculus humanus|GBMH0624-06||France|BOLD:AAA1556
Pediculus humanus corporis|GBCYB012-14
Pediculus humanus corporis|GBCYB011-14
Pediculus humanus corporis|GBCYB010-14
Pediculus humanus corporis|GBMNF34400-22||Iraq|BOLD:AAA1556
Pediculus humanus capitis|GBMNE91879-22||Iraq|BOLD:AAA1556
Pediculus humanus capitis|GBMHP120-15|taxon:121226|Thailand|BOLD:AAA1556
Pediculus humanus|GBMH1077-06||United States
Pediculus humanus|GBMH1078-06||United States
Pediculus humanus capitis|GBMNB60224-20|||BOLD:AAA1556
Pediculus humanus capitis|GBMHP272-15|taxon:121226|Thailand|BOLD:AAA1556
Pediculus humanus capitis|GBMHP273-15|taxon:121226|Thailand|BOLD:AAA1556
Pediculus humanus capitis|GBMHP111-15|taxon:121226|Thailand|BOLD:AAA1556
Pediculus humanus capitis|GBMHP112-15|taxon:121226|Thailand|BOLD:AAA1556
Pediculus humanus capitis|GBMHP113-15|taxon:121226|Thailand|BOLD:AAA1556
Pediculus humanus capitis|GBMHP117-15|taxon:121226|Thailand|BOLD:AAA1556
Pediculus humanus capitis|GBMHP121-15|taxon:121226|Thailand|BOLD:AAA1556
Pediculus humanus capitis|GBMHP124-15|taxon:121226|Thailand|BOLD:AAA1556
Pediculus humanus capitis|GBMHP131-15|taxon:121226|Thailand|BOLD:AAA1556
Pediculus humanus capitis|GBMHP135-15|taxon:121226|Thailand|BOLD:AAA1556
Pediculus humanus capitis|GBMHP140-15|taxon:121226|Thailand|BOLD:AAA1556
Pediculus humanus capitis|GBMHP142-15|taxon:121226|Thailand|BOLD:AAA1556
Pediculus humanus capitis|GBMHP176-15|taxon:121226|Thailand|BOLD:AAA1556
Pediculus humanus capitis|GBMHP182-15|taxon:121226|Thailand|BOLD:AAA1556
Pediculus humanus capitis|GBMHP183-15|taxon:121226|Thailand|BOLD:AAA1556
Pediculus humanus capitis|GBMHP186-15|taxon:121226|Thailand|BOLD:AAA1556
Pediculus humanus capitis|GBMHP187-15|taxon:121226|Thailand|BOLD:AAA1556
Pediculus humanus capitis|GBMHP188-15|taxon:121226|Thailand|BOLD:AAA1556
Pediculus humanus capitis|GBMHP189-15|taxon:121226|Thailand|BOLD:AAA1556
Pediculus humanus capitis|GBMHP191-15|taxon:121226|Thailand|BOLD:AAA1556
Pediculus humanus capitis|GBMHP196-15|taxon:121226|Thailand|BOLD:AAA1556
Pediculus humanus capitis|GBMHP199-15|taxon:121226|Thailand|BOLD:AAA1556
Pediculus humanus capitis|GBMHP206-15|taxon:121226|Thailand|BOLD:AAA1556
Pediculus humanus capitis|GBMHP214-15|taxon:121226|Thailand|BOLD:AAA1556
Pediculus humanus capitis|GBMHP215-15|taxon:121226|Thailand|BOLD:AAA1556
Pediculus humanus capitis|GBMHP216-15|taxon:121226|Thailand|BOLD:AAA1556
Pediculus humanus capitis|GBMHP218-15|taxon:121226|Thailand|BOLD:AAA1556
Pediculus humanus capitis|GBMHP220-15|taxon:121226|Thailand|BOLD:AAA1556
Pediculus humanus capitis|GBMHP221-15|taxon:121226|Thailand|BOLD:AAA1556
Pediculus humanus capitis|GBMHP222-15|taxon:121226|Thailand|BOLD:AAA1556
Pediculus humanus capitis|GBMHP228-15|taxon:121226|Thailand|BOLD:AAA1556
Pediculus humanus capitis|GBMHP230-15|taxon:121226|Thailand|BOLD:AAA1556
Pediculus humanus capitis|GBMHP235-15|taxon:121226|Thailand|BOLD:AAA1556
Pediculus humanus capitis|GBMHP241-15|taxon:121226|Thailand|BOLD:AAA1556
Pediculus humanus capitis|GBMHP242-15|taxon:121226|Thailand|BOLD:AAA1556
Pediculus humanus capitis|GBMHP243-15|taxon:121226|Thailand|BOLD:AAA1556
Pediculus humanus capitis|GBMHP248-15|taxon:121226|Thailand|BOLD:AAA1556
Pediculus humanus capitis|GBMHP256-15|taxon:121226|Thailand|BOLD:AAA1556
Pediculus humanus capitis|GBMHP259-15|taxon:121226|Thailand|BOLD:AAA1556
Pediculus humanus capitis|GBMHP290-15|taxon:121226|Thailand|BOLD:AAA1556
Pediculus humanus capitis|GBMHP292-15|taxon:121226|Thailand|BOLD:AAA1556
Pediculus humanus capitis|GBMHP293-15|taxon:121226|Thailand|BOLD:AAA1556
Pediculus humanus capitis|GBMHP310-15|taxon:121226|Thailand|BOLD:AAA1556
Pediculus humanus capitis|GBMHP311-15|taxon:121226|Thailand|BOLD:AAA1556
Pediculus humanus capitis|GBMHP313-15|taxon:121226|Thailand|BOLD:AAA1556
Pediculus humanus capitis|GBMHP314-15|taxon:121226|Thailand|BOLD:AAA1556
Pediculus humanus capitis|GBMHP316-15|taxon:121226|Thailand|BOLD:AAA1556
Pediculus humanus capitis|GBMHP319-15|taxon:121226|Thailand|BOLD:AAA1556
Pediculus humanus capitis|GBMHP322-15|taxon:121226|Thailand|BOLD:AAA1556
Pediculus humanus capitis|GBMHP333-15|taxon:121226|Thailand|BOLD:AAA1556
Pediculus humanus capitis|GBMHP337-15|taxon:121226|Thailand|BOLD:AAA1556
Pediculus humanus capitis|GBMHP339-15|taxon:121226|Thailand|BOLD:AAA1556
Pediculus humanus capitis|GBMHP346-15|taxon:121226|Thailand|BOLD:AAA1556
Pediculus humanus capitis|GBMHP348-15|taxon:121226|Thailand|BOLD:AAA1556
Pediculus humanus capitis|GBMNE24057-21|||BOLD:AAA1556
Pediculus humanus capitis|GBMHP115-15|taxon:121226|Thailand|BOLD:AAA1556
Pediculus humanus capitis|GBMHP177-15|taxon:121226|Thailand|BOLD:AAA1556
Pediculus humanus capitis|GBMHP180-15|taxon:121226|Thailand|BOLD:AAA1556
Pediculus humanus|GBMH5453-09
Pediculus humanus|GBMH1053-06||Canada
Pediculus humanus|GBCYB041-14||Australia|BOLD:AAA1556
Pediculus humanus capitis|GBMNB60230-20||Pakistan
Pediculus humanus|GBCYB039-14||Australia|BOLD:AAA1556
Pediculus humanus corporis|GBMHP105-14|||BOLD:AAA1556
Pediculus humanus capitis|GBCYB014-14|||BOLD:AAA1556
Pediculus humanus capitis|GBCYB017-14|||BOLD:AAA1556
Pediculus humanus|GBCYB038-14||Australia|BOLD:AAA1556
Pediculus humanus|GBCYB037-14||Australia|BOLD:AAA1556

Pediculus humanus capitis|GBCYB017-14|||BOLD:AAA1556
Pediculus humanus|GBCYB038-14||Australia|BOLD:AAA1556
Pediculus humanus|GBCYB037-14||Australia|BOLD:AAA1556
Pediculus humanus corporis|GBCYB030-14|||BOLD:AAA1556
Pediculus humanus capitis|GBCYB027-14|||BOLD:AAA1556
Pediculus humanus corporis|GBCYB020-14|||BOLD:AAA1556
Pediculus humanus corporis|GBCYB016-14|||BOLD:AAA1556
Pediculus humanus capitis|GBCYB013-14|||BOLD:AAA1556
Pediculus humanus capitis|GBMHP023-13|||BOLD:AAA1556
Pediculus humanus|GBMH5497-09|||BOLD:AAA1556
Pediculus humanus|GBMH1585-06|||BOLD:AAA1556
Pediculus humanus corporis|GBCYB032-14|||BOLD:AAA1556
Pediculus humanus capitis|MLICE005-18|Egg, Found on hair of mummy|Iran|BOLD:AAA1556
Pediculus humanus|GBCYB040-14||Australia|BOLD:AAA1556
Pediculus humanus|ENTJR349-08||Thailand|BOLD:AAA1556
Pediculus humanus|ENTJR346-08||Thailand|BOLD:AAA1556
Pediculus humanus corporis|GBMHP103-14|||BOLD:AAA1556
Pediculus humanus|ENTJR348-08||Thailand|BOLD:AAA1556
Pediculus humanus|ENTJR342-08||Thailand|BOLD:AAA1556
Pediculus humanus|ENTJR341-08||Thailand|BOLD:AAA1556
Pediculus humanus corporis|GBMHP104-14|||BOLD:AAA1556
Pediculus humanus capitis|GBMHP303-15|taxon:121226|Thailand|BOLD:AAA1556
Pediculus humanus capitis|GBMHP304-15|taxon:121226|Thailand|BOLD:AAA1556
Pediculus humanus capitis|MLICE004-18|Egg, Found on hair of mummy|Iran
Pediculus humanus capitis|MLICE007-18|Egg, Found on hair of mummy|Iran
Pediculus humanus corporis|GBMNF34391-22||Iraq|BOLD:AAA1556
Pediculus humanus corporis|GBMNF34393-22||Iraq|BOLD:AAA1556
Pediculus humanus corporis|GBMNF34397-22||Iraq|BOLD:AAA1556
Pediculus humanus corporis|GBMNF34398-22||Iraq|BOLD:AAA1556
Pediculus humanus corporis|GBMNF34399-22||Iraq|BOLD:AAA1556
Pediculus humanus corporis|GBCYB028-14|||BOLD:AAA1556
Pediculus humanus corporis|GBMNF34394-22||Iraq|BOLD:AAA1556
Pediculus humanus capitis|GBCYB023-14|||BOLD:AAA1556
Pediculus humanus corporis|GBCYB024-14|||BOLD:AAA1556
Pediculus humanus capitis|GBCYB025-14|||BOLD:AAA1556
Pediculus humanus corporis|GBCYB026-14|||BOLD:AAA1556
Pediculus humanus corporis|GBMNF34395-22||Iraq|BOLD:AAA1556
Pediculus humanus corporis|GBMNF34401-22||Iraq|BOLD:AAA1556
Pediculus humanus corporis|GBMNF34392-22||Iraq|BOLD:AAA1556
Pediculus humanus corporis|GBMNF34396-22||Iraq|BOLD:AAA1556
Pediculus humanus corporis|GBMNF34402-22||Iraq|BOLD:AAA1556
Pediculus humanus capitis|GBMNA25514-19|||BOLD:AAA1556
Pediculus humanus capitis|GBCYB043-14||Australia|BOLD:AAA1556
Pediculus humanus capitis|GBCYB042-14||Australia|BOLD:AAA1556
Pediculus humanus corporis|GBCYB019-14|||BOLD:AAA1556
Pediculus humanus capitis|JSBIC118-17||Canada|BOLD:AAA1556
Pediculus humanus capitis|JSBIC119-17||Canada|BOLD:AAA1556
Pediculus humanus|MLICE021-19|Egg on hair of person currently living in Iran|Iran|BOLD:AAA1556
Pediculus humanus capitis|FILOU246-19||Finland|BOLD:AAA1556
Pediculus humanus|MLICE017-19|Egg on hair of person currently living in Iran|Iran|BOLD:AAA1556
Pediculus humanus|MLICE018-19|Egg on hair of person currently living in Iran|Iran|BOLD:AAA1556
Pediculus humanus|MLICE019-19|Egg on hair of person currently living in Iran|Iran|BOLD:AAA1556
Pediculus humanus|MLICE020-19|Egg on hair of person currently living in Iran|Iran|BOLD:AAA1556
Pediculus humanus|MLICE022-19|Egg on hair of person currently living in Iran|Iran|BOLD:AAA1556
Pediculus humanus capitis|PAVEA080-22||Austria|BOLD:AAA1556
Pediculus humanus capitis|GBMNE91871-22||Iraq|BOLD:AAA1556
Pediculus humanus capitis|GBMNE91872-22||Iraq|BOLD:AAA1556
Pediculus humanus capitis|GBMNE91876-22||Iraq|BOLD:AAA1556
Pediculus humanus capitis|GBMNE91877-22||Iraq|BOLD:AAA1556
Pediculus humanus capitis|GBMNE91878-22||Iraq|BOLD:AAA1556
Pediculus humanus capitis|GBMNE91873-22||Iraq|BOLD:AAA1556
Pediculus humanus capitis|GBMNE91874-22||Iraq|BOLD:AAA1556
Pediculus humanus capitis|GBMNE91880-22||Iraq|BOLD:AAA1556
Pediculus humanus capitis|GBMNE91870-22||Iraq|BOLD:AAA1556
Pediculus humanus capitis|GBMNE91875-22||Iraq|BOLD:AAA1556
Pediculus humanus capitis|GBMNE91881-22||Iraq|BOLD:AAA1556
Pediculus humanus|GBMH0973-06||Nepal
Pediculus humanus|GBMH0972-06||Nepal
Pediculus humanus|GBMH0971-06||Nepal
Pediculus humanus|GBMH0970-06||Nepal
Pediculus humanus|GBMH0969-06||Nepal
Pediculus humanus|GBMH0968-06||Nepal
Pediculus humanus|GBMH0967-06||Nepal
Pediculus humanus|GBMH0966-06||Nepal
Pediculus humanus|GBMH0965-06||Nepal
Pediculus humanus|GBMH0964-06||Nepal
Pediculus humanus|GBMH0948-06||Nepal
Pediculus humanus|GBMH0947-06||Nepal
Pediculus humanus|GBMH5277-09||United States|BOLD:AAA1556
Pediculus humanus|GBMH1082-06||Honduras|BOLD:AAA1556
Pediculus humanus|GBMH1079-06||Philippines|BOLD:AAA1556
Pediculus humanus|GBMH1016-06||Nepal
Pediculus humanus|GBMH1017-06||Nepal
Pediculus humanus|GBMH1010-06||Argentina
Pediculus humanus|GBMH1011-06||Brazil
Pediculus humanus|GBMH1012-06||Brazil
Pediculus humanus|GBMH5333-09||Argentina|BOLD:AAA1556
Pediculus humanus|GBMH5452-09|||BOLD:AAA1556
Pediculus humanus|GBMH0946-06||Nepal
Pediculus humanus|GBMH0945-06||China
Pediculus humanus|GBMH0959-06||Iran
Pediculus humanus|GBMH0960-06||Iran
Pediculus humanus|GBMH0996-06||Nepal
Pediculus humanus|GBMH0995-06||China
Pediculus humanus|GBMH0994-06||China
Pediculus humanus|GBMH0958-06||Nepal
Pediculus humanus|GBMH0957-06||China
Pediculus humanus|GBMH0956-06||China
[illegible]

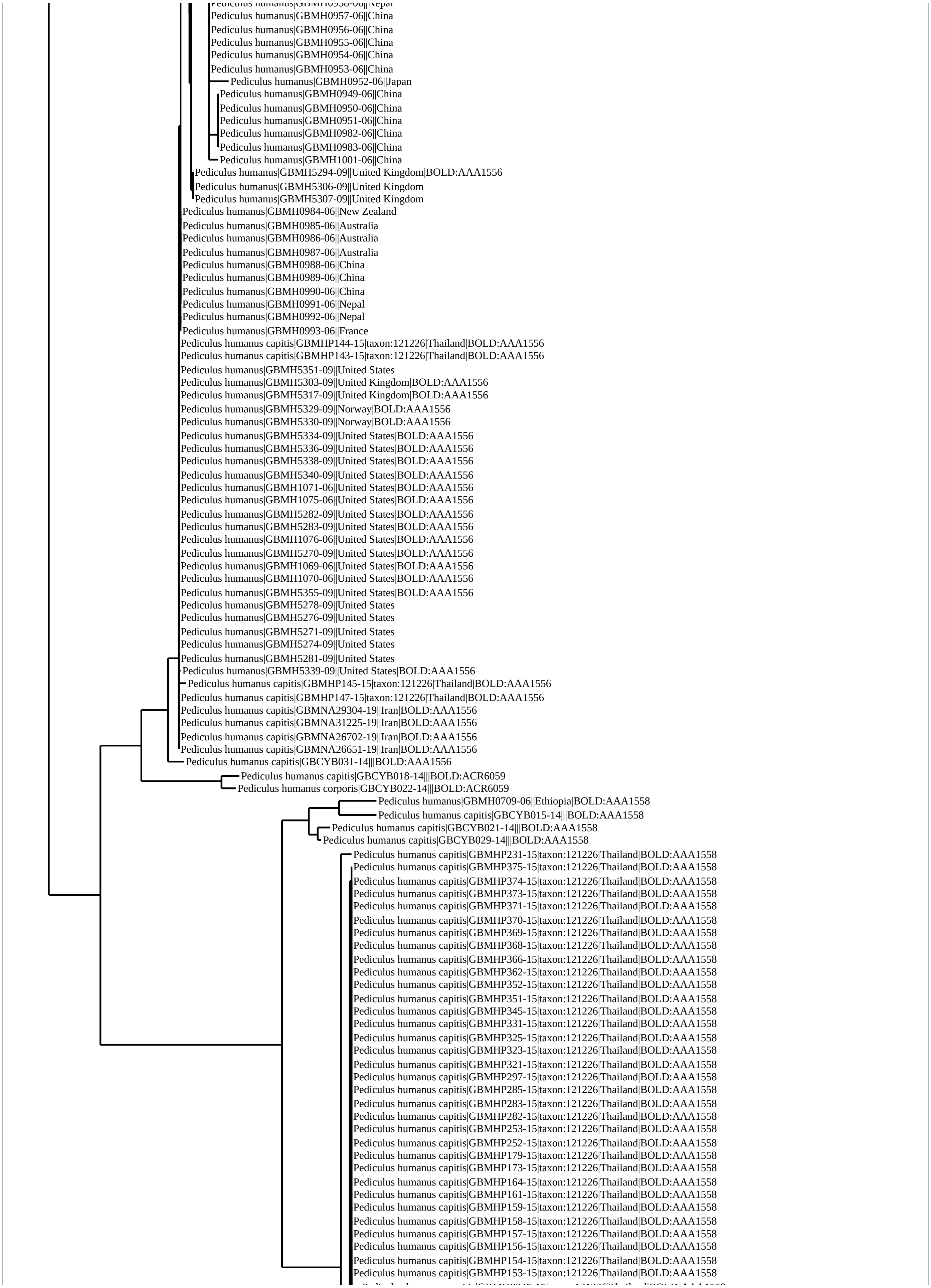
Pediculus humanus|GBMH0958-06||Nepal
Pediculus humanus|GBMH0957-06||China
Pediculus humanus|GBMH0956-06||China
Pediculus humanus|GBMH0955-06||China
Pediculus humanus|GBMH0954-06||China
Pediculus humanus|GBMH0953-06||China
Pediculus humanus|GBMH0952-06||Japan
Pediculus humanus|GBMH0949-06||China
Pediculus humanus|GBMH0950-06||China
Pediculus humanus|GBMH0951-06||China
Pediculus humanus|GBMH0982-06||China
Pediculus humanus|GBMH0983-06||China
Pediculus humanus|GBMH1001-06||China
Pediculus humanus|GBMH5294-09||United Kingdom|BOLD:AAA1556
Pediculus humanus|GBMH5306-09||United Kingdom
Pediculus humanus|GBMH5307-09||United Kingdom
Pediculus humanus|GBMH0984-06||New Zealand
Pediculus humanus|GBMH0985-06||Australia
Pediculus humanus|GBMH0986-06||Australia
Pediculus humanus|GBMH0987-06||Australia
Pediculus humanus|GBMH0988-06||China
Pediculus humanus|GBMH0989-06||China
Pediculus humanus|GBMH0990-06||China
Pediculus humanus|GBMH0991-06||Nepal
Pediculus humanus|GBMH0992-06||Nepal
Pediculus humanus|GBMH0993-06||France
Pediculus humanus capitis|GBMHP144-15|taxon:121226|Thailand|BOLD:AAA1556
Pediculus humanus capitis|GBMHP143-15|taxon:121226|Thailand|BOLD:AAA1556
Pediculus humanus|GBMH5351-09||United States
Pediculus humanus|GBMH5303-09||United Kingdom|BOLD:AAA1556
Pediculus humanus|GBMH5317-09||United Kingdom|BOLD:AAA1556
Pediculus humanus|GBMH5329-09||Norway|BOLD:AAA1556
Pediculus humanus|GBMH5330-09||Norway|BOLD:AAA1556
Pediculus humanus|GBMH5334-09||United States|BOLD:AAA1556
Pediculus humanus|GBMH5336-09||United States|BOLD:AAA1556
Pediculus humanus|GBMH5338-09||United States|BOLD:AAA1556
Pediculus humanus|GBMH5340-09||United States|BOLD:AAA1556
Pediculus humanus|GBMH1071-06||United States|BOLD:AAA1556
Pediculus humanus|GBMH1075-06||United States|BOLD:AAA1556
Pediculus humanus|GBMH5282-09||United States|BOLD:AAA1556
Pediculus humanus|GBMH5283-09||United States|BOLD:AAA1556
Pediculus humanus|GBMH1076-06||United States|BOLD:AAA1556
Pediculus humanus|GBMH5270-09||United States|BOLD:AAA1556
Pediculus humanus|GBMH1069-06||United States|BOLD:AAA1556
Pediculus humanus|GBMH1070-06||United States|BOLD:AAA1556
Pediculus humanus|GBMH5355-09||United States|BOLD:AAA1556
Pediculus humanus|GBMH5278-09||United States
Pediculus humanus|GBMH5276-09||United States
Pediculus humanus|GBMH5271-09||United States
Pediculus humanus|GBMH5274-09||United States
Pediculus humanus|GBMH5281-09||United States
Pediculus humanus|GBMH5339-09||United States|BOLD:AAA1556
Pediculus humanus capitis|GBMHP145-15|taxon:121226|Thailand|BOLD:AAA1556
Pediculus humanus capitis|GBMHP147-15|taxon:121226|Thailand|BOLD:AAA1556
Pediculus humanus capitis|GBMNA29304-19||Iran|BOLD:AAA1556
Pediculus humanus capitis|GBMNA31225-19||Iran|BOLD:AAA1556
Pediculus humanus capitis|GBMNA26702-19||Iran|BOLD:AAA1556
Pediculus humanus capitis|GBMNA26651-19||Iran|BOLD:AAA1556
Pediculus humanus capitis|GBCYB031-14|||BOLD:AAA1556
Pediculus humanus capitis|GBCYB018-14|||BOLD:ACR6059
Pediculus humanus corporis|GBCYB022-14|||BOLD:ACR6059
Pediculus humanus|GBMH0709-06||Ethiopia|BOLD:AAA1558
Pediculus humanus capitis|GBCYB015-14|||BOLD:AAA1558
Pediculus humanus capitis|GBCYB021-14|||BOLD:AAA1558
Pediculus humanus capitis|GBCYB029-14|||BOLD:AAA1558
Pediculus humanus capitis|GBMHP231-15|taxon:121226|Thailand|BOLD:AAA1558
Pediculus humanus capitis|GBMHP375-15|taxon:121226|Thailand|BOLD:AAA1558
Pediculus humanus capitis|GBMHP374-15|taxon:121226|Thailand|BOLD:AAA1558
Pediculus humanus capitis|GBMHP373-15|taxon:121226|Thailand|BOLD:AAA1558
Pediculus humanus capitis|GBMHP371-15|taxon:121226|Thailand|BOLD:AAA1558
Pediculus humanus capitis|GBMHP370-15|taxon:121226|Thailand|BOLD:AAA1558
Pediculus humanus capitis|GBMHP369-15|taxon:121226|Thailand|BOLD:AAA1558
Pediculus humanus capitis|GBMHP368-15|taxon:121226|Thailand|BOLD:AAA1558
Pediculus humanus capitis|GBMHP366-15|taxon:121226|Thailand|BOLD:AAA1558
Pediculus humanus capitis|GBMHP362-15|taxon:121226|Thailand|BOLD:AAA1558
Pediculus humanus capitis|GBMHP352-15|taxon:121226|Thailand|BOLD:AAA1558
Pediculus humanus capitis|GBMHP351-15|taxon:121226|Thailand|BOLD:AAA1558
Pediculus humanus capitis|GBMHP345-15|taxon:121226|Thailand|BOLD:AAA1558
Pediculus humanus capitis|GBMHP331-15|taxon:121226|Thailand|BOLD:AAA1558
Pediculus humanus capitis|GBMHP325-15|taxon:121226|Thailand|BOLD:AAA1558
Pediculus humanus capitis|GBMHP323-15|taxon:121226|Thailand|BOLD:AAA1558
Pediculus humanus capitis|GBMHP321-15|taxon:121226|Thailand|BOLD:AAA1558
Pediculus humanus capitis|GBMHP297-15|taxon:121226|Thailand|BOLD:AAA1558
Pediculus humanus capitis|GBMHP285-15|taxon:121226|Thailand|BOLD:AAA1558
Pediculus humanus capitis|GBMHP283-15|taxon:121226|Thailand|BOLD:AAA1558
Pediculus humanus capitis|GBMHP282-15|taxon:121226|Thailand|BOLD:AAA1558
Pediculus humanus capitis|GBMHP253-15|taxon:121226|Thailand|BOLD:AAA1558
Pediculus humanus capitis|GBMHP252-15|taxon:121226|Thailand|BOLD:AAA1558
Pediculus humanus capitis|GBMHP179-15|taxon:121226|Thailand|BOLD:AAA1558
Pediculus humanus capitis|GBMHP173-15|taxon:121226|Thailand|BOLD:AAA1558
Pediculus humanus capitis|GBMHP164-15|taxon:121226|Thailand|BOLD:AAA1558
Pediculus humanus capitis|GBMHP161-15|taxon:121226|Thailand|BOLD:AAA1558
Pediculus humanus capitis|GBMHP159-15|taxon:121226|Thailand|BOLD:AAA1558
Pediculus humanus capitis|GBMHP158-15|taxon:121226|Thailand|BOLD:AAA1558
Pediculus humanus capitis|GBMHP157-15|taxon:121226|Thailand|BOLD:AAA1558
Pediculus humanus capitis|GBMHP156-15|taxon:121226|Thailand|BOLD:AAA1558
Pediculus humanus capitis|GBMHP154-15|taxon:121226|Thailand|BOLD:AAA1558
Pediculus humanus capitis|GBMHP153-15|taxon:121226|Thailand|BOLD:AAA1558

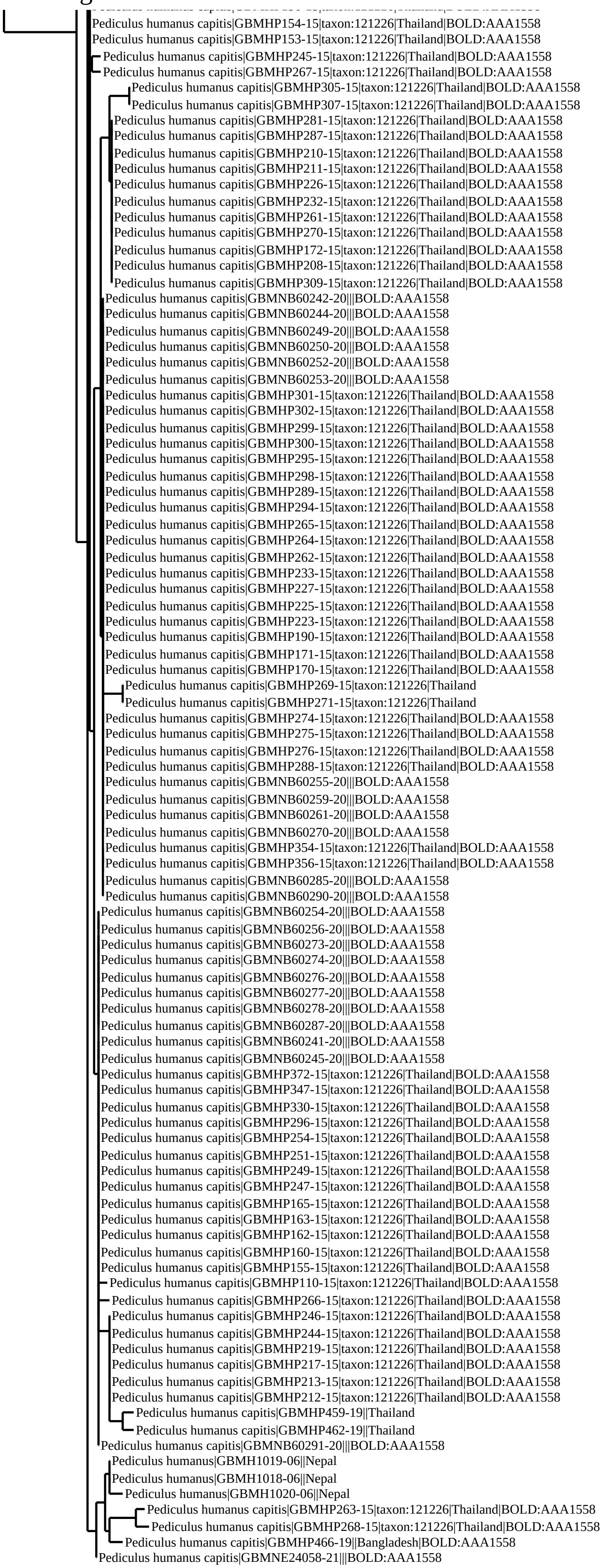
Pediculus humanus capitis|GBMHP154-15|taxon:121226|Thailand|BOLD:AAA1558
Pediculus humanus capitis|GBMHP153-15|taxon:121226|Thailand|BOLD:AAA1558
Pediculus humanus capitis|GBMHP245-15|taxon:121226|Thailand|BOLD:AAA1558
Pediculus humanus capitis|GBMHP267-15|taxon:121226|Thailand|BOLD:AAA1558
Pediculus humanus capitis|GBMHP305-15|taxon:121226|Thailand|BOLD:AAA1558
Pediculus humanus capitis|GBMHP307-15|taxon:121226|Thailand|BOLD:AAA1558
Pediculus humanus capitis|GBMHP281-15|taxon:121226|Thailand|BOLD:AAA1558
Pediculus humanus capitis|GBMHP287-15|taxon:121226|Thailand|BOLD:AAA1558
Pediculus humanus capitis|GBMHP210-15|taxon:121226|Thailand|BOLD:AAA1558
Pediculus humanus capitis|GBMHP211-15|taxon:121226|Thailand|BOLD:AAA1558
Pediculus humanus capitis|GBMHP226-15|taxon:121226|Thailand|BOLD:AAA1558
Pediculus humanus capitis|GBMHP232-15|taxon:121226|Thailand|BOLD:AAA1558
Pediculus humanus capitis|GBMHP261-15|taxon:121226|Thailand|BOLD:AAA1558
Pediculus humanus capitis|GBMHP270-15|taxon:121226|Thailand|BOLD:AAA1558
Pediculus humanus capitis|GBMHP172-15|taxon:121226|Thailand|BOLD:AAA1558
Pediculus humanus capitis|GBMHP208-15|taxon:121226|Thailand|BOLD:AAA1558
Pediculus humanus capitis|GBMHP309-15|taxon:121226|Thailand|BOLD:AAA1558
Pediculus humanus capitis|GBMNB60242-20|||BOLD:AAA1558
Pediculus humanus capitis|GBMNB60244-20|||BOLD:AAA1558
Pediculus humanus capitis|GBMNB60249-20|||BOLD:AAA1558
Pediculus humanus capitis|GBMNB60250-20|||BOLD:AAA1558
Pediculus humanus capitis|GBMNB60252-20|||BOLD:AAA1558
Pediculus humanus capitis|GBMNB60253-20|||BOLD:AAA1558
Pediculus humanus capitis|GBMHP301-15|taxon:121226|Thailand|BOLD:AAA1558
Pediculus humanus capitis|GBMHP302-15|taxon:121226|Thailand|BOLD:AAA1558
Pediculus humanus capitis|GBMHP299-15|taxon:121226|Thailand|BOLD:AAA1558
Pediculus humanus capitis|GBMHP300-15|taxon:121226|Thailand|BOLD:AAA1558
Pediculus humanus capitis|GBMHP295-15|taxon:121226|Thailand|BOLD:AAA1558
Pediculus humanus capitis|GBMHP298-15|taxon:121226|Thailand|BOLD:AAA1558
Pediculus humanus capitis|GBMHP289-15|taxon:121226|Thailand|BOLD:AAA1558
Pediculus humanus capitis|GBMHP294-15|taxon:121226|Thailand|BOLD:AAA1558
Pediculus humanus capitis|GBMHP265-15|taxon:121226|Thailand|BOLD:AAA1558
Pediculus humanus capitis|GBMHP264-15|taxon:121226|Thailand|BOLD:AAA1558
Pediculus humanus capitis|GBMHP262-15|taxon:121226|Thailand|BOLD:AAA1558
Pediculus humanus capitis|GBMHP233-15|taxon:121226|Thailand|BOLD:AAA1558
Pediculus humanus capitis|GBMHP227-15|taxon:121226|Thailand|BOLD:AAA1558
Pediculus humanus capitis|GBMHP225-15|taxon:121226|Thailand|BOLD:AAA1558
Pediculus humanus capitis|GBMHP223-15|taxon:121226|Thailand|BOLD:AAA1558
Pediculus humanus capitis|GBMHP190-15|taxon:121226|Thailand|BOLD:AAA1558
Pediculus humanus capitis|GBMHP171-15|taxon:121226|Thailand|BOLD:AAA1558
Pediculus humanus capitis|GBMHP170-15|taxon:121226|Thailand|BOLD:AAA1558
Pediculus humanus capitis|GBMHP269-15|taxon:121226|Thailand
Pediculus humanus capitis|GBMHP271-15|taxon:121226|Thailand
Pediculus humanus capitis|GBMHP274-15|taxon:121226|Thailand|BOLD:AAA1558
Pediculus humanus capitis|GBMHP275-15|taxon:121226|Thailand|BOLD:AAA1558
Pediculus humanus capitis|GBMHP276-15|taxon:121226|Thailand|BOLD:AAA1558
Pediculus humanus capitis|GBMHP288-15|taxon:121226|Thailand|BOLD:AAA1558
Pediculus humanus capitis|GBMNB60255-20|||BOLD:AAA1558
Pediculus humanus capitis|GBMNB60259-20|||BOLD:AAA1558
Pediculus humanus capitis|GBMNB60261-20|||BOLD:AAA1558
Pediculus humanus capitis|GBMNB60270-20|||BOLD:AAA1558
Pediculus humanus capitis|GBMHP354-15|taxon:121226|Thailand|BOLD:AAA1558
Pediculus humanus capitis|GBMHP356-15|taxon:121226|Thailand|BOLD:AAA1558
Pediculus humanus capitis|GBMNB60285-20|||BOLD:AAA1558
Pediculus humanus capitis|GBMNB60290-20|||BOLD:AAA1558
Pediculus humanus capitis|GBMNB60254-20|||BOLD:AAA1558
Pediculus humanus capitis|GBMNB60256-20|||BOLD:AAA1558
Pediculus humanus capitis|GBMNB60273-20|||BOLD:AAA1558
Pediculus humanus capitis|GBMNB60274-20|||BOLD:AAA1558
Pediculus humanus capitis|GBMNB60276-20|||BOLD:AAA1558
Pediculus humanus capitis|GBMNB60277-20|||BOLD:AAA1558
Pediculus humanus capitis|GBMNB60278-20|||BOLD:AAA1558
Pediculus humanus capitis|GBMNB60287-20|||BOLD:AAA1558
Pediculus humanus capitis|GBMNB60241-20|||BOLD:AAA1558
Pediculus humanus capitis|GBMNB60245-20|||BOLD:AAA1558
Pediculus humanus capitis|GBMHP372-15|taxon:121226|Thailand|BOLD:AAA1558
Pediculus humanus capitis|GBMHP347-15|taxon:121226|Thailand|BOLD:AAA1558
Pediculus humanus capitis|GBMHP330-15|taxon:121226|Thailand|BOLD:AAA1558
Pediculus humanus capitis|GBMHP296-15|taxon:121226|Thailand|BOLD:AAA1558
Pediculus humanus capitis|GBMHP254-15|taxon:121226|Thailand|BOLD:AAA1558
Pediculus humanus capitis|GBMHP251-15|taxon:121226|Thailand|BOLD:AAA1558
Pediculus humanus capitis|GBMHP249-15|taxon:121226|Thailand|BOLD:AAA1558
Pediculus humanus capitis|GBMHP247-15|taxon:121226|Thailand|BOLD:AAA1558
Pediculus humanus capitis|GBMHP165-15|taxon:121226|Thailand|BOLD:AAA1558
Pediculus humanus capitis|GBMHP163-15|taxon:121226|Thailand|BOLD:AAA1558
Pediculus humanus capitis|GBMHP162-15|taxon:121226|Thailand|BOLD:AAA1558
Pediculus humanus capitis|GBMHP160-15|taxon:121226|Thailand|BOLD:AAA1558
Pediculus humanus capitis|GBMHP155-15|taxon:121226|Thailand|BOLD:AAA1558
Pediculus humanus capitis|GBMHP110-15|taxon:121226|Thailand|BOLD:AAA1558
Pediculus humanus capitis|GBMHP266-15|taxon:121226|Thailand|BOLD:AAA1558
Pediculus humanus capitis|GBMHP246-15|taxon:121226|Thailand|BOLD:AAA1558
Pediculus humanus capitis|GBMHP244-15|taxon:121226|Thailand|BOLD:AAA1558
Pediculus humanus capitis|GBMHP219-15|taxon:121226|Thailand|BOLD:AAA1558
Pediculus humanus capitis|GBMHP217-15|taxon:121226|Thailand|BOLD:AAA1558
Pediculus humanus capitis|GBMHP213-15|taxon:121226|Thailand|BOLD:AAA1558
Pediculus humanus capitis|GBMHP212-15|taxon:121226|Thailand|BOLD:AAA1558
Pediculus humanus capitis|GBMHP459-19||Thailand
Pediculus humanus capitis|GBMHP462-19||Thailand
Pediculus humanus capitis|GBMNB60291-20|||BOLD:AAA1558
Pediculus humanus|GBMH1019-06||Nepal
Pediculus humanus|GBMH1018-06||Nepal
Pediculus humanus|GBMH1020-06||Nepal
Pediculus humanus capitis|GBMHP263-15|taxon:121226|Thailand|BOLD:AAA1558
Pediculus humanus capitis|GBMHP268-15|taxon:121226|Thailand|BOLD:AAA1558
Pediculus humanus capitis|GBMHP466-19||Bangladesh|BOLD:AAA1558
Pediculus humanus capitis|GBMNE24058-21|||BOLD:AAA1558
